# A new contribution to *Megasporoporia* sensu lato: Six new species and three new combinations

**DOI:** 10.3389/fmicb.2022.1046777

**Published:** 2022-12-08

**Authors:** Ya-Rong Wang, Yu-Cheng Dai, Hong-Gao Liu, Josef Vlasák, Peter Buchanan, Yuan Yuan, Ying-Da Wu

**Affiliations:** ^1^Institute of Microbiology, School of Ecology and Nature Conservation, Beijing Forestry University, Beijing, China; ^2^School of Agronomy and Life Sciences, Zhaotong University, Zhaotong, China; ^3^Biology Centre, Czech Academy of Sciences, Institute of Plant Molecular Biology, Ceské Budějovice, Czechia; ^4^Manaaki Whenua - Landcare Research, Auckland, New Zealand; ^5^Key Laboratory of Forest and Grassland Fire Risk Prevention, Ministry of Emergency Management, China Fire and Rescue Institute, Beijing, China

**Keywords:** taxonomy, phylogeny, morphology, Polyporaceae, polymerase

## Abstract

*Megasporoporia* sensu lato has recently been intensively studied in China and South America, and four independent clades representing four genera have been recognized phylogenetically. In this study, more samples, mostly from subtropical and tropical Asia, Oceania, and East Africa, are analyzed. A phylogeny based on a 4-gene dataset of sequences (ITS + nLSU + mtSSU + *tef*) has confirmed the presence of four genera in *Megasporoporia* sensu lato: *Jorgewrightia, Mariorajchenbergia, Megasporia*, and *Megasporoporia* sensu stricto. Six new species, *Jorgewrightia austroasiana, Jorgewrightia irregularis, Jorgewrightia tenuis, Mariorajchenbergia subleucoplaca, Megasporia olivacea*, and *Megasporia sinuosa*, are described based on morphology and phylogenetic analysis. Three new combinations are proposed, viz. *Jorgewrightia kirkii, Mariorajchenbergia epitephra*, and *Mariorajchenbergia leucoplaca*. To date, 36 species of *Megasporoporia* sensu lato are accepted and an identification key to these species is provided. In addition, the identification of *Dichomitus amazonicus, Dichomitus cylindrosporus*, and *Megasporoporia hexagonoides* is discussed.

## Introduction

*Megasporoporia* Ryvarden and J. E. Wright was established by Ryvarden et al. (1982). The genus is readily distinguished from other polypore genera by resupinate basidiocarps with large pores, a dimitic hyphal structure, usually the presence of hyphal pegs and dendrohyphidia, and hyaline, thin-walled, and big basidiospores. The species of the genus was found in tropical or subtropical Africa, America, and Asia (Ryvarden et al., 1982; Lira et al., 2021; Wang et al., 2021; Wu et al., 2022a). Li and Cui (2013) performed the first phylogenetic analysis of the genus based on ITS + nLSU sequences, demonstrating that *Megasporoporia* was a polyphyletic genus with three independent clades nested in *Megasporoporia* sensu lato. So, they proposed *Megasporia* B. K. Cui et al., *Megasporoporia* sensu stricto, and *Megasporoporiella* B. K. Cui et al. to represent these clades, although these three genera have similar morphology. Subsequently, Yuan et al. (2017) described three new species of *Megasporia* from China. Recently, Wang et al. (2021) made a comprehensive phylogenetic analysis based on a 4-gene dataset (ITS + nLSU + mtSSU + *tef*) from 21 species of *Megasporoporia* sensu lato, and they found a new clade, described four new species, and proposed two combinations. Lira et al. (2021) revised the definition of *Megasporoporia* sensu lato and proposed *Jorgewrightia* Gibertoni and C. R. S. Lira and *Mariorajchenbergia* Gibertoni and C. R. S. Lira. They also confirmed that the four clades nested in *Megasporoporia* sensu lato were *Jorgewrightia, Mariorajchenbergia, Megasporia*, and *Megasporoporia* sensu stricto. In addition, two new species were described and 20 combinations were proposed (Lira et al., 2021). Several new species have been confirmed recently in *Megasporoporia* sensu lato, especially from subtropical and tropical areas (Lira et al., 2021; Wang et al., 2021).

This study continues to research on the diversity and phylogeny of *Megasporoporia* sensu lato based on samples from subtropical and tropical Asia, Oceania, and East Africa. Six new species are described and three new combinations are proposed.

## Materials and methods

### Morphological studies

The voucher specimens are deposited at the herbaria of the Institute of Microbiology, Beijing Forestry University (BJFC); Universidade Federal de Pernambuco (URM); University of Oslo; the National Museum Prague of Czech Republic (PRM); and the private herbarium of Josef Vlasák (JV). Macro-morphological descriptions were based on field notes and herbarium specimens. Special color terms follow Anonymous (1969) and Petersen (1996). Microscopic analyses follow Wu et al. (2022b). The following abbreviations were used: CB = Cotton Blue, CB+ = cyanophilous in Cotton Blue, CB– = acyanophilous in Cotton Blue, IKI = Melzer's reagent, IKI– = neither amyloid nor dextrinoid, KOH = 2% potassium hydroxide, L = arithmetic average of all spore lengths, W = arithmetic average of all spore widths, Q = L/W ratios, and *n* = (a/b), where the number of spores (a) is measured from the given number of specimens (b).

### DNA extraction, amplification, and sequencing

The CTAB rapid plant genome extraction kit (Aidlab Biotechnologies Co., Ltd, Beijing) procedures were used to extract total genomic DNA from the fruiting bodies and for the polymerase chain reaction (PCR) according to the manufacturer's instructions with some modifications (Chen et al., 2016; Shen et al., 2019). The PCR primers for all genes are ITS5 (GGA AGT AAA AGT CGT AAC AAG G), ITS4 (TCC TCC GCT TAT TGA TAT GC), LR0R (ACC CGC TGA ACT TAA GC), LR7 (TAC TAC CAC CAA GAT CT), MS1 (CAG CAG TCA AGA ATA TTA GTC AAT G), MS2 (GCG GAT TAT CGA ATT AAA TAA C), 983F (GCY CCY GGH CAY CGT GAY TTY AT), and 1567R (ACH GTR CCR ATA CCA CCR ATC TT), according to White et al. (1990), Vilgalys and Hester (1990), and Rehner and Buckley (2005). The final PCR volume was 30 μl, which contained 1 μl of each primer, 1 μl extracted DNA, 12 μl ddH_2_O, and 15 μl 2 × EasyTaq PCR Supermix (TransGen Biotech Co., Ltd., Beijing, China). The PCR procedures for four genes followed Wang et al. (2021).

Nuclear ribosomal RNA genes of the known 36 species of *Megasporoporia* sensu lato were used to determine the phylogenetic position of the new species. Gene sequencing was performed at the Beijing Genomics Institute, and the newly-generated sequences were deposited in GenBank. Sequences generated for this study were aligned with additional sequences downloaded from GenBank. All newly generated sequences were deposited at GenBank (http://www.ncbi.nlm.nih.gov/) and are listed in Table 1. Clustal X (Thompson et al., 1997) and BioEdit (Hall, 1999) were used to align and collate these sequences. The data matrices were edited in Mesquite v3.04 software (Maddison and Maddison, 2021). The combined matrix was reconstructed for phylogenetic analysis as a 4-gene dataset (ITS + nLSU + mtSSU + *tef*). Sequences of *Trametes hirsuta* (Wulfen) Lloyd and *T. ochracea* (Pers.) Gilb. and Ryvarden were used as outgroups to root trees (Wang et al., 2021). The phylogenetic analysis used in this study followed the approach of Zhu et al. (2019) and Sun et al. (2020). Maximum parsimony (MP), maximum likelihood (ML), and Bayesian inference (BI) were employed to perform phylogenetic analysis.

**Table 1:** Taxa information and GenBank accession numbers of the sequences used in this study.

**Species**	**Sample no**.	**Geographic origin**	**GenBank accessions**				**References**
			**ITS**	**nLSU**	**mt-SSU**	***tef***	
*Crassisporus imbricatus*	Dai 10788	China	KC867350	KC867425	KX838374	–	Cui et al., 2019
*C. imbricatus*	Cui 6556	China	KC867351	KC867426	–	–	Cui et al., 2019
*C. leucoporus*	Cui 16801	Australia	MK116488	MK116497	MK116507	MK122986	Cui et al., 2019
*C. macroporus*	Cui 14465	China	MK116485	MK116494	MK116504	MK122983	Cui et al., 2019
*C. macroporus*	Cui 14468	China	MK116486	MK116495	MK116505	MK122984	Cui et al., 2019
*C. microsporus*	Cui 16221	China	MK116487	MK116496	MK116506	MK122985	Cui et al., 2019
*Daedaleopsis confragosa*	Cui 6892	China	KU892428	KU892448	KX838381	KX838418	Cui et al., 2019
*D. confragosa*	Cui 9756	China	KU892438	KU892451	–	–	Cui et al., 2019
*D. hainanensis*	Dai 9268	China	KU892434	KU892458	KX838414	–	Li et al., 2016
*D. hainanensis*	Cui 5178	China	KU892435	KU892462	KX838413	KX838441	Li et al., 2016
*D. purpurea*	Dai 8060	Japan	KU892442	KU892475	KX838409	KX838438	Li et al., 2016
*D. purpurea*	Dai 13583a	China	KX832054	KX832063	KX838412	KX838440	Cui et al., 2019
*Datronia mollis*	Dai 11456	China	JX559253	JX559292	KX838388	KX838424	Li et al., 2014
*D. mollis*	Dai 11253	China	JX559258	JX559289	KX838387	–	Li et al., 2014
*Datroniella subtropica*	Dai 12883	China	KC415184	KC415191	KX838390	KX838427	Li et al., 2014
*D. subtropica*	Dai 12885	China	KC415185	KC415192	KX838391	KX838428	Li et al., 2014
*Dichomitus squalens*	Cui 9639	China	JQ780407	JQ780426	KX838404	KX838436	Li and Cui, 2013
*D. squalens*	Cui 9725	China	JQ780408	JQ780427	KX838403	KX838435	Li and Cui, 2013
*D. squalens*	Cui 15870	China	**ON088330**	**ON089003**	–	–	Present study
*D. squalens*	Cui 18424	China	**ON088331**	**ON089004**	–	–	Present study
*D. squalens*	Cui 18438	China	**ON088332**	**ON089005**	–	–	Present study
*D. squalens*	Dai 15352	China	**ON088333**	**ON089006**	–	–	Present study
*Echinochaete russiceps*	Dai 13868	China	KX832051	KX832060	KX838406	KX838437	Cui et al., 2019
*E. russiceps*	Dai 13866	China	KX832050	KX832059	KX838405	–	Cui et al., 2019
*Favolus acervatus*	Cui 11053	China	KU189774	KU189805	KU189956	KU189920	Zhou and Cui, 2017
*F. acervatus*	Dai 10749b	China	KX548953	KX548979	KX549018	KX549043	Zhou and Cui, 2017
*F. niveus*	Cui 11129	China	KX548955	KX548981	KX549019	KX549045	Zhou and Cui, 2017
*F. niveus*	Dai 13276	China	KX548956	KX548982	KX549020	KX549046	Zhou and Cui, 2017
*F. pseudoemerici*	Cui 11079	China	KX548958	KX548984	KX549022	KX549048	Zhou and Cui, 2017
*F. pseudoemerici*	Cui 13757	China	KX548959	KX548985	KX549023	KX549049	Zhou and Cui, 2017
*Hexagonia glabra*	Dai 12993	China	KX900637	KX900683	KX900733	KX900823	Cui et al., 2019
*H. glabra*	Cui 11367	China	KX900638	KX900684	KX900734	KX900824	Cui et al., 2019
*Hornodermoporus latissimus*	Cui 6625	China	HQ876604	JF706340	KF051040	KF181134	Zhao and Cui, 2012
*H. latissimus*	Dai 12054	China	KX900639	KX900686	KF218297	KF286303	Cui et al., 2019
***Jorgewrightia austroasiana***	**Dai 17884 (holotype)**	**Singapore**	**MW422260**	**ON089007**	**ON088341**	**–**	Present study
***J. austroasiana***	**Dai 18627**	**Malaysia**	**MW422261**	**ON089008**	**–**	**–**	Present study
*J. bambusae*	Dai 22106 (holotype)	China	MW694884	–	MW694912	MZ618631	Wang et al., 2021
*J. bambusae*	Dai 20064	China	MW694885	MW694928	MW694913	MZ618632	Wang et al., 2021
*J. cystidiolophora*	Cui 2642	China	JQ780390	JQ780432	–	–	Li and Cui, 2013
*J. cystidiolophora*	Cui 2688 (paratype)	China	JQ780389	JQ780431	–	–	Li and Cui, 2013
*J. ellipsoidea*	Dai 19743	China	MW694879	MW694923	MW694899	MZ669220	Wang et al., 2021
*J. ellipsoidea*	Cui 5222 (holotype)	China	JQ314367	JQ314390	–	–	Li and Cui, 2013
*J. fusiformis*	Dai 18596 (holotype)	Malaysia	MW694892	MW694935	MW694920	MZ618637	Wang et al., 2021
*J. fusiformis*	Dai 18578	Malaysia	MW694893	MW694936	MW694921	MZ618638	Wang et al., 2021
*J. guangdongensis*	Cui 9130 (holotype)	China	JQ314373	JQ780428	–	–	Li and Cui, 2013
*J. guangdongensis*	Cui 13986	China	MG847208	MG847217	MG847229	MG867699	Cui et al., 2019
*J. hengduanensis*	Cui 8076 (holotype)	China	JQ780392	JQ780433	MG847252	KF286337	Li and Cui, 2013
*J. hengduanensis*	Cui 8176	China	JQ314370	KX900697	KX900749	MG867700	Li and Cui, 2013
***J. kirkii***	**Ryvarden 32577**	**Zimbabwe**	**ON088326**	**ON089001**	–	**ON158685**	Present study
*J. major*	Cui 10253	China	JQ314366	JQ780437	MK116502	–	Li and Cui, 2013
*J. major*	Yuan 1183	China	JQ314365	–	–	–	Li and Cui, 2013
*J. rimosa*	Dai 15357 (holotype)	China	KY449436	KY449447	MW694908	–	Yuan et al., 2017
*J. rimosa*	Dai 21997	China	MW422262	–	MW694909	–	Wang et al., 2021
***J. irregularis***	**Dai 16449**	**China**	**ON088318**	**ON088994**	**–**	–	Present study
***J. irregularis***	**Cui 13853 (holotype)**	**China**	MW694880	MW694924	MW694900	MZ618625	Wang et al., 2021
***J. tenuis***	**Dai 20510 (holotype)**	**China**	**ON088323**	**ON088998**	**ON088338**	–	Present study
***J. tenuis***	**Dai 20517**	**China**	**ON088324**	**ON088999**	**ON088339**	**ON158684**	Present study
*J. tropica*	Cui 13740	China	KY449438	KY449449	MW694910	MZ618629	Yuan et al., 2017
*J. tropica*	Cui 13660 (holotype)	China	KY449437	KY449448	MW694911	MZ618630	Yuan et al., 2017
*J. violacea*	Cui 13845	China	MG847211	MG847220	MG847232	MG867703	Cui et al., 2019
*J. violacea*	Cui 13838	China	MG847210	MG847219	MG847231	MG867702	Cui et al., 2019
*J. yunnanensis*	Cui 12614A	China	KY449442	KY449453	MW694922	MZ618628	Yuan et al., 2017
*J. yunnanensis*	Dai 13870 (holotype)	China	KY449443	KY449454	MW694907	–	Yuan et al., 2017
***M. epitephra***	**Coveny 219**	**Australia**	**ON088325**	**ON089000**	–	–	Present study
*M. hubeiensis*	Dai 18102	China	MW694890	MW694933	MW694918	MZ618636	Wang et al., 2021
*M. hubeiensis*	Dai 18103	China	MW694891	MW694934	MW694919	–	Wang et al., 2021
*Mariorajchenbergia leucoplaca*	Dai 18657 (holotype of *Megasporoporiella australiae*)	Australia	MW694888	MW694931	MW694916	MZ618634	Wang et al., 2021
*M. leucoplaca*	Dai 18658	Australia	MW694889	MW694932	MW694917	MZ618635	Wang et al., 2021
*M. leucoplaca*	ICMP 16412	New Zealand	ON944162	ON944142			Present study
*M. leucoplaca*	ICMP 16962	New Zealand	ON944161	ON944141			Present study
*M. leucoplaca*	ICMP 17545	New Zealand	ON944160	ON944140			Present study
*M. pseudocavernulosa*	Yuan 1270 (holotype)	China	JQ314360	JQ314394	–	–	Li and Cui, 2013
*M. pseudocavernulosa*	Dai 19379	China	MW694882	–	MW694904	MZ618626	Wang et al., 2021
*M. rhododendri*	Dai 4226 (holotype)	China	JQ314356	JQ314392	MW694905	–	Li and Cui, 2013
*M. rhododendri*	Cui 12432	China	MW694883	MW694927	MW694906	MZ618627	Wang et al., 2021
*M. subcavernulosa*	Cui 9252	China	JQ780378	JQ780416	MG847235	MG867706	Li and Cui, 2013
*M. subcavernulosa*	Cui 14247	China	MG847213	MG847222	MG847234	MG867705	Cui et al., 2019
***M. subleucoplaca***	**Ryvarden 11049 (holotype)**	**Tanzania**	**ON088327**	–	–	–	Present study
*Megasporia amazonia*	URM 87859	Brazil	MW989394	MW965595	–	–	Wang et al., 2021
*M. amazonia*	URM 85601	Brazil	KX584455	KX619579	–	MW161494	Lira et al., 2021
*M. anoectopora*	URM 86947	Brazil	KX584456	KX619577	–	MW045831	Lira et al., 2021
*M. anoectopora*	URM 86928	Brazil	KX584457	KX619580	–	MW161495	Lira et al., 2021
*M. cavernulosa*	URM 83867	Brazil	KX584458	KX619582	–	–	Lira et al., 2021
*M. hexagonoides*	CBS 464.63	Argentina	–	AY333802	–	–	Lira et al., 2021
*M. mexicana*	JV 1806/4-J	Honduras	MW989396	–	–	–	Wang et al., 2021
***M. olivacea***	**Dai 17908 (holotype)**	**China**	**ON088328**	**ON089002**	**–**	**ON158686**	Present study
***M. olivacea***	**Dai 17909**	**China**	**ON088329**	–	**ON088340**	**ON158687**	Present study
***M. sinuosa***	**Dai 22011**	**China**	**ON088321**	**ON088996**	**ON088336**	**ON158682**	Present study
***M. sinuosa***	**Dai 22210 (holotype)**	**China**	**ON088322**	**ON088997**	**ON088337**	**ON158683**	Present study
*M*. sp. 1	JV 0904/81	USA	MW989395	–	–	–	Wang et al., 2021
*M*. sp. 1	JV 0904/52-J	USA	JF894107	**ON088995**	**ON088335**	**ON158681**	Present study
*M*. sp. 1	JV 0904/50-J	USA	JF894105	–	–	–	Present study
*M. variabilicolor*	URM 88369	Brazil	KX584449	KX619578	–	MW045833	Lira et al., 2021
*M. variabilicolor*	URM 88368	Brazil	KX584448	KX619574	–	MW161496	Lira et al., 2021
*Megasporoporia bannaensis*	Dai 12306 (holotype)	China	JQ314362	JQ314379	–	–	Li and Cui, 2013
*M. bannaensis*	Dai 13596	China	KX900653	KX900702	KX900754	KX900838	Cui et al., 2019
*M. inflata*	Dai 17882	Malaysia	MW694886	MW694929	MW694914	–	Wang et al., 2021
*M. inflata*	Dai 17478 (holotype)	Malaysia	MW694887	MW694930	MW694915	MZ618633	Wang et al., 2021
*M. minor*	Dai 18322	Vietnam	MW694881	MW694925	MW694901	MZ618624	Wang et al., 2021
*M. minor*	Dai 12170 (holotype)	China	JQ314363	JQ314380	MW694902	KF494980	Li and Cui, 2013
*M. neosetulosa*	JV 1008/51-J	USA	JF894109	–	–	–	Li and Cui, 2013
*M. neosetulosa*	JV 1008/102-J	USA	JF894110	–	–	–	Li and Cui, 2013
*M. neosetulosa*	URM 85679 (holotype)	Brazil	KX584459	OL684780	–	–	Lira et al., 2021
*M. neosetulosa*	URM 85113	Brazil	KX584460	–	–	MW045832	Lira et al., 2021
*M. neosetulosa*	JV 0904/139-J	USA	**ON088320**	–	–	–	Present study
*M. setulosa*	LR 9907 (neotype)	Tanzania	OL678508	OL684781	–	–	Lira et al., 2021
*Neodatronia gaoligongensis*	Cui 8055	China	JX559269	JX559286	MG847236	KX900846	Li et al., 2014
*N. gaoligongensis*	Cui 8186	China	JX559268	JX559285	MG847237	–	Li et al., 2014
*Perenniporia martia*	Cui 4055	China	KX900641	KX900688	KX900737	–	Cui et al., 2019
*P. martia*	Cui 7992	China	HQ876603	HQ654114	KF051041	KF181135	Zhao and Cui, 2012
*Polyporus tuberaster*	Dai 12462	China	KU507580	KU507582	KU507584	KU507590	Zhou et al., 2016
*P. tuberaster*	Dai 11271	China	KU189769	KU189800	KU189950	KU189914	Zhou et al., 2016
*P. varius*	Cui 12249	China	KU507581	KU507583	KU507585	KU507591	Zhou et al., 2016
*P. varius*	Dai 13874	China	KU189777	KU189808	KU189958	KU189923	Zhou et al., 2016
*Trametes hirsuta*	RLG 5133T	USA	JN164941	JN164801	–	JN164891	Li and Cui, 2013
*T. ochracea*	HHB 13445sp	USA	JN164954	JN164812	–	JN164904	Li and Cui, 2013

New taxa and new sequences are in bold.

Maximum Parsimony and bootstrap values (MP-BS) obtained from 1,000 replicates were performed using PAUP^*^ version 4.0b10 (Swofford, 2002). All characters were equally weighted, and the gaps were treated as missing data. Trees were inferred using the heuristic search option with TBR branch swapping and 1,000 random sequence additions. Max trees were set to 5,000, branches of zero length were collapsed, and all parsimonious trees were saved. Clade robustness was assessed using bootstrap analysis with 1,000 replicates (Felsenstein, 1985). Descriptive tree statistics tree length (TL), consistency index (CI), retention index (RI), rescaled consistency index (RC), and homoplasy index (HI) were calculated for each maximum parsimonious tree generated (Farris, 1989; Farris et al., 1994).

Maximum likelihood (ML) was conducted with RAxML-HPC v. 8.2.3 (Stamatakis, 2014), involving 1,000 ML searches under the GTRGAMMA model, and only the maximum likelihood best tree from all searches was kept. In addition, 1,000 rapid bootstrap replicates were run with the GTRCAT model to assess the ML BS values (ML) of the nodes.

MrMODELTEST v. 2.3 (Posada and Crandall, 1998; Nylander, 2004) also was used to determine the best-fit evolution model for the combined datasets of ITS + nLSU and ITS + nLSU + mtSSU + *tef* sequences for estimating BI. BI was performed using MrBayes v. 3.2.6 (Ronquist and Huelsenbeck, 2003; Ronquist et al., 2012) with four simultaneous independent chains for two datasets, where 2 million generations were performed until the split deviation frequency value of <0.01 and were sampled every 100th generation. The first 25% of sampled trees were discarded as burn-in, while the remaining ones were used to calculate the Bayesian Posterior Probabilities (BPP) of the clades.

**Figure 1:**
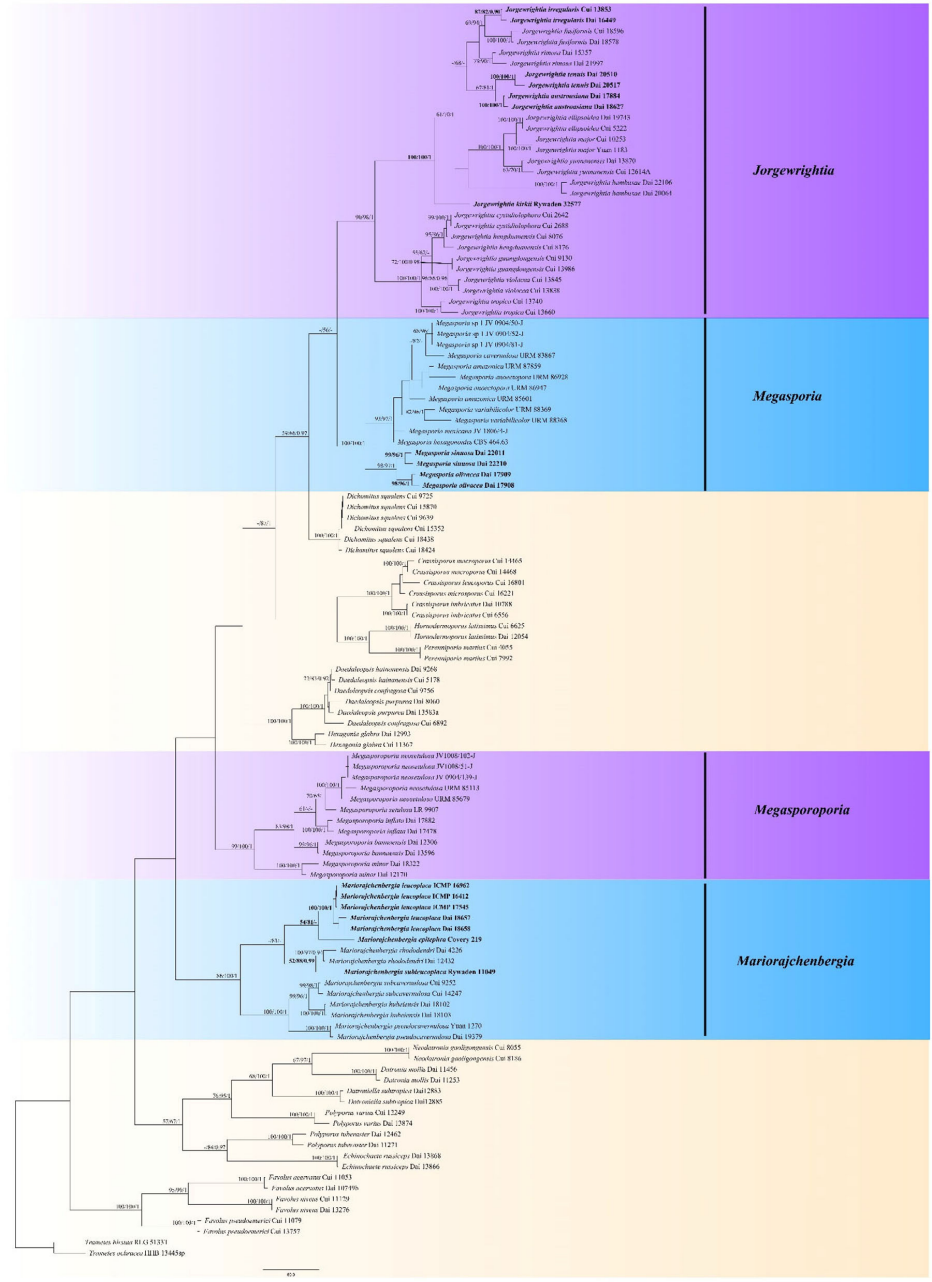
Phylogeny of *Megasporoporia* sensu lato and related species generated by Maximum Parsimony (MP) based on combined ITS + nLSU + mtSSU + *tef* sequences. Bootstrap supports for Maximum Parsimony (MP), Maximum Likelihood (ML), and Bayesian Posterior Probabilities (BPP) were not lower than 50% (MP and ML) and 0.90 (BPP) on the branches. The new species and new combinations are in bold.

Branches that received bootstrap support for MP, ML, and BPP more than or equal to 50% (MP and ML) and 0.90 (BPP) were considered significantly supported (Figure 1). The phylogenetic tree was visualized with the program FigTree v. 1.4.3 (http://tree.bio.ed.ac.uk/software/figtree/).

## Results

### Phylogenetic analysis

Analyses were based on a combined dataset of 4-gene (ITS + nLSU + mtSSU + *tef*) sequences from 114 fungal collections representing 58 species. According to MrMODELTEST v.2.3, the most suitable model was GTR + I + G, lset nst = 6, rates = invgamma, and prset statefreqpr = Dirichlet (1,1,1,1). The alignment length of the dataset of each tree generated by MP analysis is 3,548 characters, of which 2,238 characters are constant, 1,146 characters are parsimony-informative, and other key data are TL = 5,018, CI = 0.392, RI = 0.772, RC = 0.302, and HI = 0.608. BI analysis generated a congruent topology with an average standard deviation of split frequencies = 0.007589 for MP and ML analyses. Thus, the topology from the MP tree is presented along with statistical values from the MP/ML/BPP algorithms (Figure 1).

The phylogeny shows that the samples of *Megasporoporia* sensu lato form *four* clades (Figure 1): *Jorgewrightia* (90% MP, 98% ML, 1.00 BPP), *Mariorajchenbergia* (86% MP, 100% ML, 1.00 BPP), *Megasporia* (100% MP, 100% ML, 1.00 BPP), and *Megasporoporia* sensu stricto (99% MP, 100% ML, 1.00 BPP).

Fifteen lineages are nested in the *Jorgewrightia* clade. Among them, three new lineages represent three new species: *J. austroasiana* sp. nov. (100% MP, 100% ML, 1.00 BPP), *J. irregularis* sp. nov. (87% MP, 82% ML, 0.90 BPP), and *J. tenuis* sp. nov. (100% MP, 100% ML, 1.00 BPP), and another lineage represents the taxon *J. kirkii* comb. nov. (100% MP, 100% ML, 1.00 BPP).

Seven lineages are nested in the *Mariorajchenbergia* clade. Among them, one lineage represents *M. subleucoplaca* sp. nov. (52% MP, 88% ML, 0.99 BPP), and two lineages represent *M. epitephra* comb. nov. (54% MP, 81% ML) and *M. leucoplaca* comb. nov. (100% MP, 100% ML, 1.00 BPP).

Nine lineages are nested in the *Megasporia* clade, and among them, two new lineages represent *M. olivacea* sp. nov. (98% MP, 96% ML, 1.00 BPP) and *M. sinuosa* sp. nov. (99% MP, 96% ML, 1.00 BPP).

Five lineages are nested in the *Megasporoporia* sensu stricto clade.

### Taxonomy

**Figure 2:**
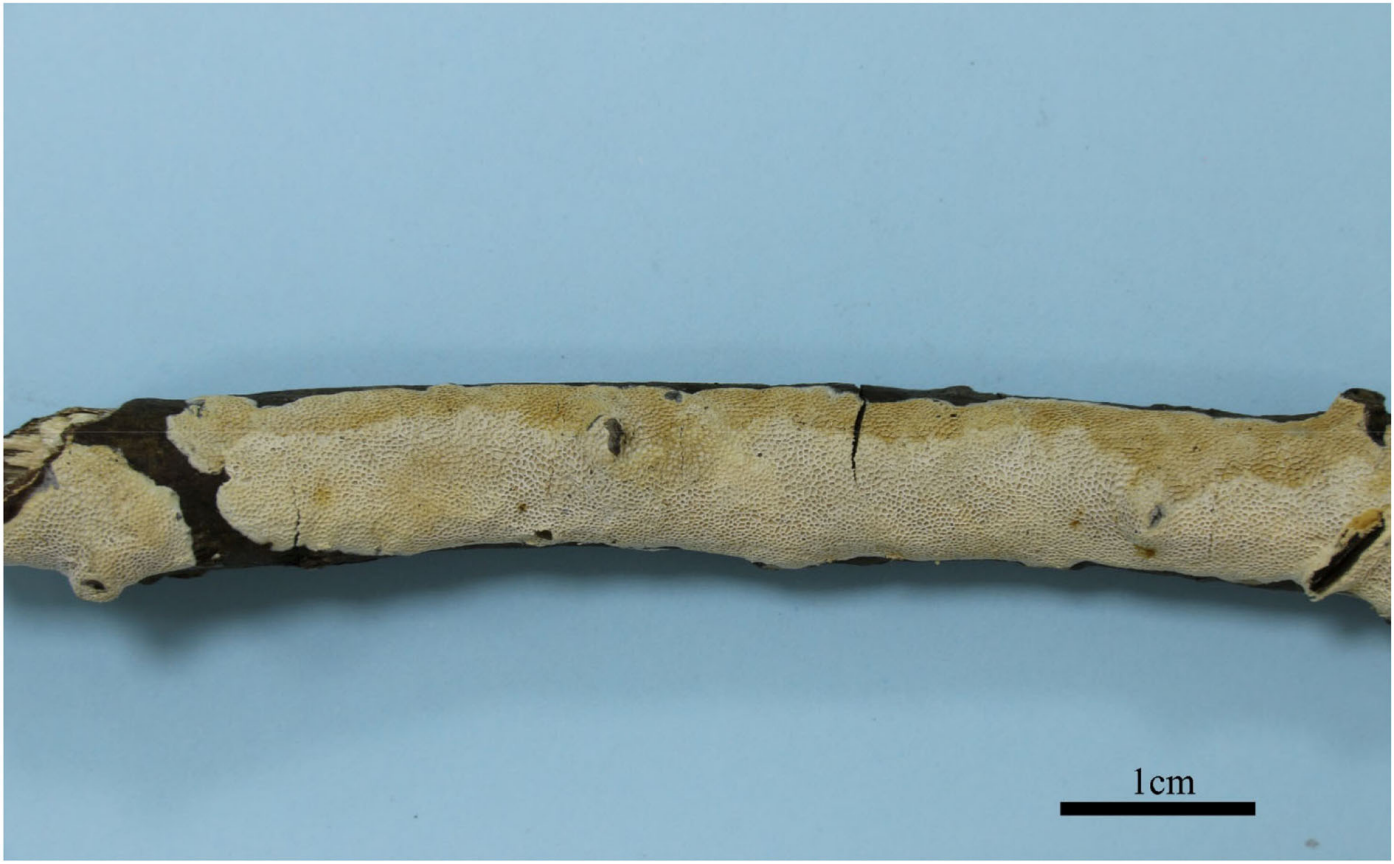
Basidiocarps of *Jorgewrightia austroasiana* (holotype, Dai 17884).

**Figure 3:**
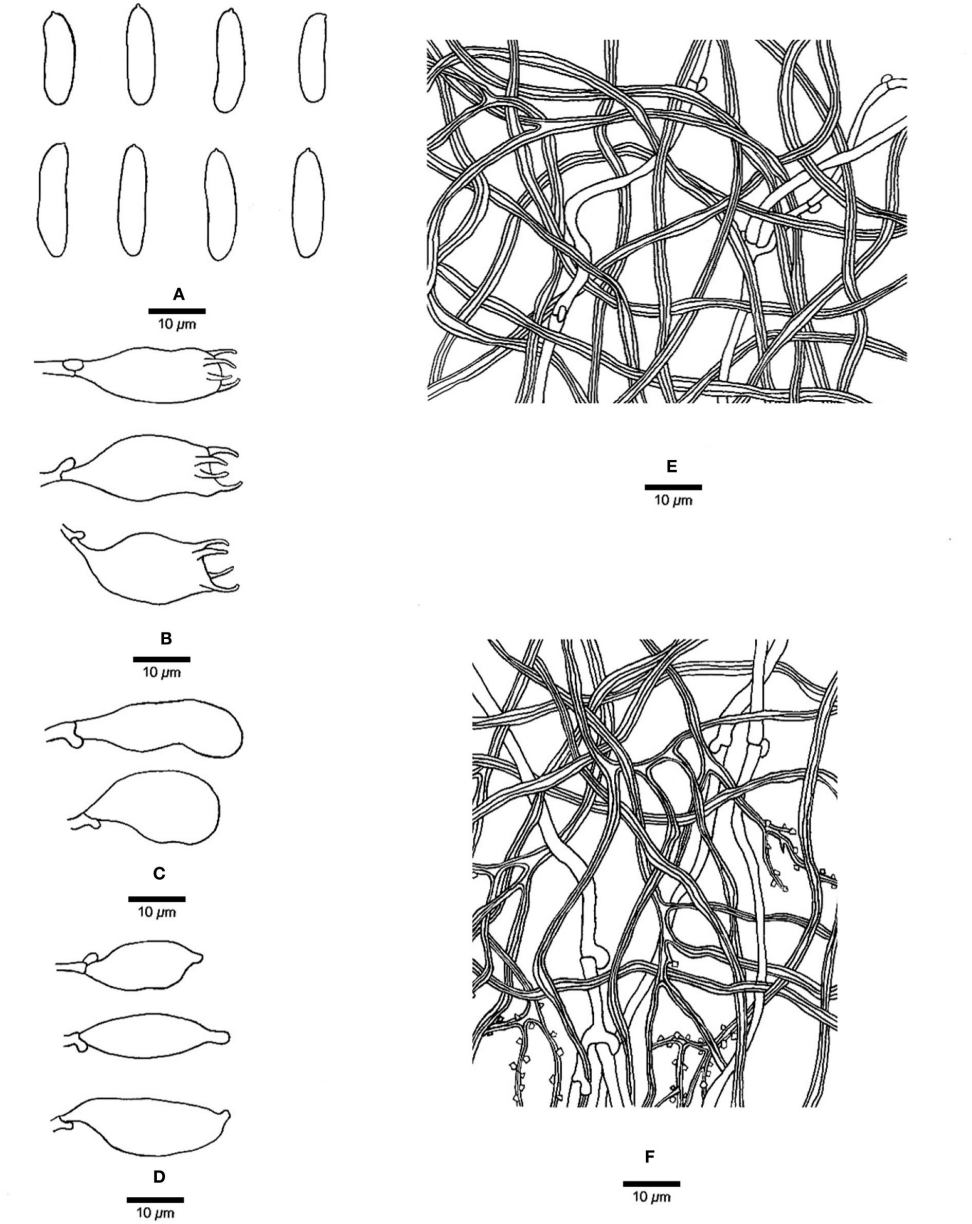
Microscopic structures of *Jorgewrightia austroasiana* (drawn from the holotype, Dai 17884). **(A)** basidiospores, **(B)** basidia, **(C)** basidioles, **(D)** cystidioles, **(E)** hyphae from subiculum, and **(F)** hyphae from tube trama.

***Jorgewrightia austroasiana*** Y. C. Dai, Yuan, Ya R. Wang and Y. D. Wu, sp. nov. Figures 2, 3

MycoBank: 845309

*Type:* Singapore, Bukit Timah Nature Reserve, on a fallen angiosperm branch, 20 July 2017, Dai 17884 (holotype, BJFC025416!).

*Etymology: austroasiana* (Lat.) refers to the species being found in South Asia.

*Basidiocarps* annual, resupinate, adnate, corky, without odor or taste when fresh, becoming hard corky upon drying, up to 13 cm long, 2 cm wide, and 0.6 mm thick at the center; sterile margin thinning out, white when fresh, cream when dry, up to 1 mm wide. Pore surface white to cream when fresh, cream to buff when dry; pores round to angular, 3–3.5 per mm; dissepiments thick, entire; subiculum cream, corky, up to 0.2 mm thick; tubes cream, paler than subiculum, corky, up to 0.4 mm long. *Hyphal system* dimitic; generative hyphae with clamp connections; skeletal hyphae indextrinoid, CB+; tissues unchanged in KOH (not swollen). *Subicular* generative hyphae infrequent, hyaline, thin-walled, occasionally branched, 1.5–2 μm diameter; skeletal hyphae dominant, distinctly thick-walled with a narrow to medium lumen, occasionally branched, interwoven, 1.5–2.5 μm diameter. *Tramal* generative hyphae hyaline, thin-walled, occasionally branched, 1.5–3 μm diameter; skeletal hyphae dominant, thick-walled with a narrow lumen, frequently branched, mostly flexuous, interwoven, sometimes encrusted by crystals, 2–3.5 μm diameter. *Dendrohyphidia* absent. *Hyphal pegs* absent. *Cystidia* absent; *cystidioles* present, fusoid, thin-walled, smooth, 14–30 × 6.5–10 μm. *Basidia* more or less barrel-shaped, with four sterigmata and a basal clamp connection, 24–28 × 9–12 μm; basidioles in shape similar to basidia. Small tetrahedric or polyhedric crystals frequent among hymenium and trama. *Basidiospores* cylindrical, slightly curved, hyaline, thin-walled, smooth, IKI–, CB–, (14.5–) 15.0–19.5 (−20.0) × (3.2–) 3.5–6.0 (−6.5) μm, L = 16.27 μm, W = 4.50 μm, Q =3.54–3.74 (*n* = 60/2).

*Additional materials (paratypes) examined:* Malaysia, Selangor, Kota Damansara, Community Forest Reserve, on a fallen angiosperm branch, 17 April 2018, Dai 18627 (BJFC026915!).

*Notes:* Phylogenetically, *Jorgewrightia austroasiana* is related to *J. rimosa, J, tenuis, J. fusiformis*, and *J. irregularis* (Figure 1). However, *J. rimosa* differs from *J. austroasiana* by its dextrinoid skeletal hyphae and the presence of dendrohyphidia (Yuan et al., 2017). *J. fusiformis* and *J. tenuis* are readily distinguished from *J. austroasiana* by their fusiform basidiospores (Wang et al., 2021), *J. irregularis* differs from *J. austroasiana* by its bigger pores (0.5–1 per mm vs. 3–3.5 per mm) and the presence of dendrohyphidia and hyphal pegs.

**Figure 4:**
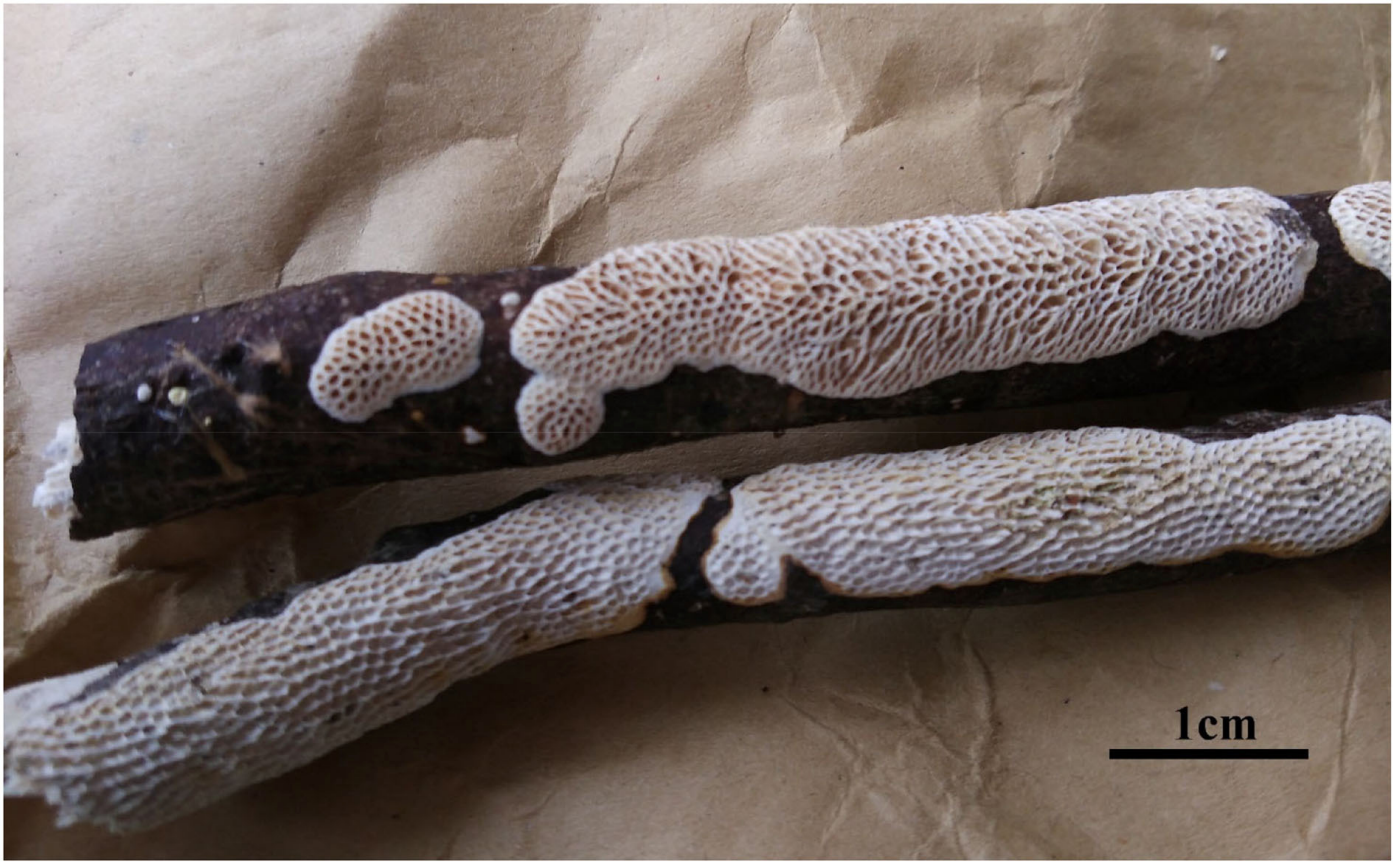
Basidiocarps of *Jorgewrightia irregularis* (Dai 16449).

**Figure 5:**
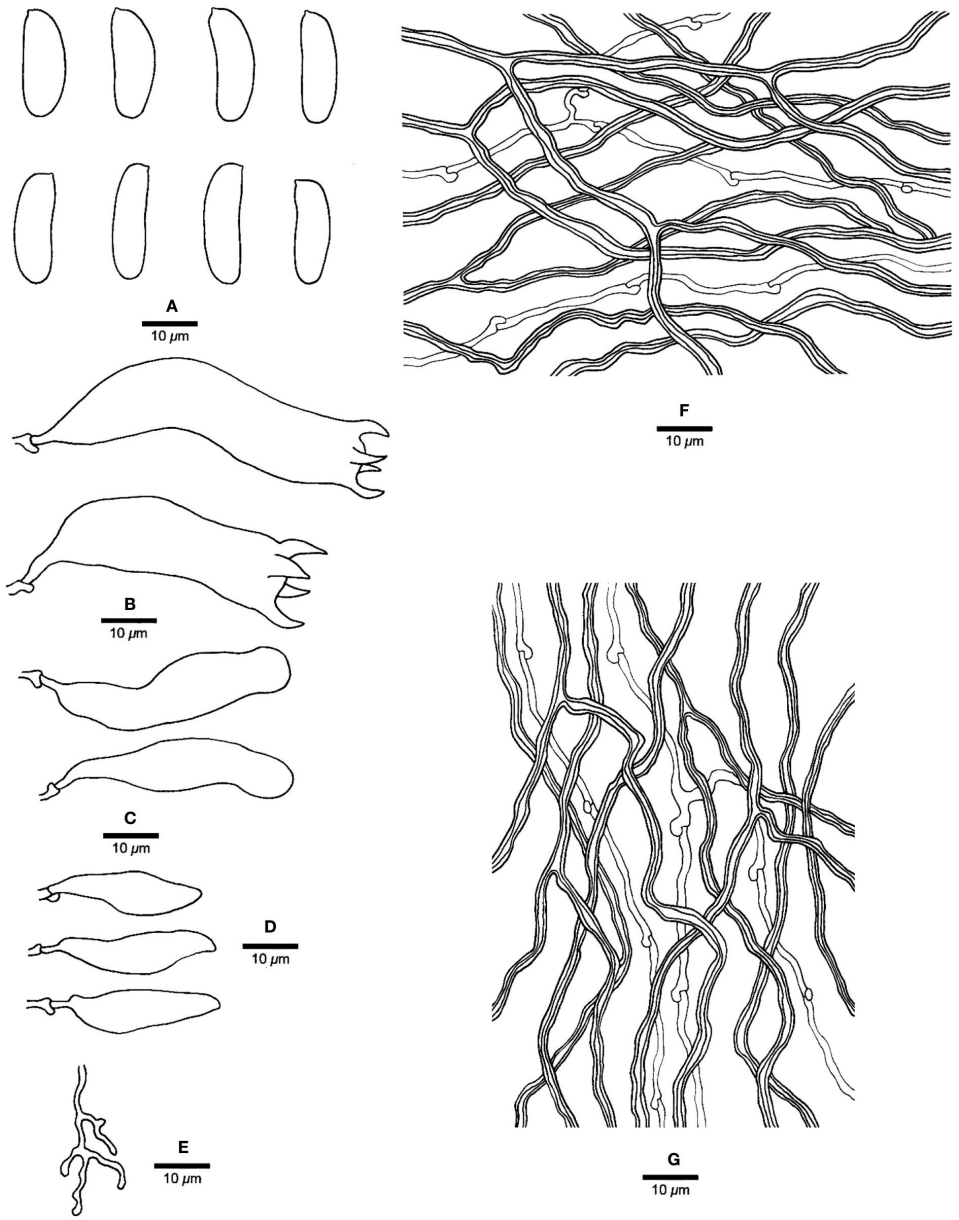
Microscopic structures of *Jorgewrightia irregularis* (drawn from holotype, Dai 13853). **(A)** basidiospores, **(B)** basidia, **(C)** basidioles, **(D)** cystidioles, **(E)** dendrohyphidia, **(F)** hyphae from subiculum, and **(G)** hyphae from tube trama.

***Jorgewrightia irregularis*** Y. C. Dai, Yuan, Ya R. Wang and Y. D. Wu, sp. nov. Figures 4, 5

MycoBank: 845312

*Type:* China, Hainan Prov., Baisha County, Yinggeling Nature Reserve, on a fallen angiosperm branch, 17 June 2016, Cui 13853 (holotype, BJFC028719!).

*Etymology: irregularis* (Lat.) refers to irregular pores of the basidiocarps.

*Basidiocarps* annual, resupinate, adnate, without odor or taste when fresh, becoming hard corky upon drying, up to 6 cm long, 1.5 cm wide, and 1.3 mm thick at the center; sterile margin cream when juvenile, brownish with age, up to 0.6 mm wide. Pore surface white to cream when fresh, buff to honey when dry; pores angular when juvenile, irregular with age, e.g., hexagonoid, sinuous or split, 0.5–1 per mm; dissepiments thick, entire to lacerate; subiculum pale cream, corky, up to 0.4 mm long; tubes buff, corky, up to 0.9 mm long. *Hyphal system* dimitic; generative hyphae with clamp connections; skeletal hyphae indextrinoid, CB+; tissues unchanged in KOH (not swollen). *Subicular* generative hyphae infrequent, hyaline, thin-walled, moderately branched, 1–1.2 μm diameter; skeletal hyphae dominant, thick-walled with a narrow to medium lumen, moderately branched, strongly flexuous, interwoven, 2.5–3 μm diameter. *Tramal* generative hyphae infrequent, hyaline, thin-walled, moderately branched, 1.2–1.5 μm diameter; skeletal hyphae dominant, thick-walled with a narrow lumen, moderately branched, strongly flexuous, interwoven, 2.2–2.5 μm diameter. *Dendrohyphidia* present. *Hyphal pegs* present. *Cystidia* absent; *cystidioles* present, subulate or ventricose, thin-walled, smooth, 25.5–32.5 × 6.5–8 μm. *Basidia* more or less clavate, usually constricted in middle, with four sterigmata and a basal clamp connection, 42–51 × 8–13 μm; *basidioles* in shape similar to basidia, but distinctly smaller. Small tetrahedric or polyhedric crystals present among hymenium and trama. *Basidiospores* cylindrical, slightly curved, hyaline, thin-walled, smooth, IKI–, CB–, (17–) 17.5–21.2 (−21.5) × (4.5–) 5–6.2 (−6.5) μm, *L* = 19.22 μm, *W* = 5.86 μm, *Q* = 3.22–3.34 (*n* = 60/2).

*Additional materials (paratypes) examined:* China, Hainan Prov., Ledong County, Jianfengling Nature Reserve, 11 May 2009, Cui 6592 (BJFC004445!); Qiongzhong County, Limushan Forest Park, on a fallen angiosperm branch, 8 June 2016, Dai 16449 (BJFC022566!).

*Notes:* Phylogenetically, *Jorgewrightia irregularis* is related to *J. fusiformis* (Figure 1), but the latter has fusiform basidiospores and lacks hyphal pegs (Wang et al., 2021).

**Figure 6:**
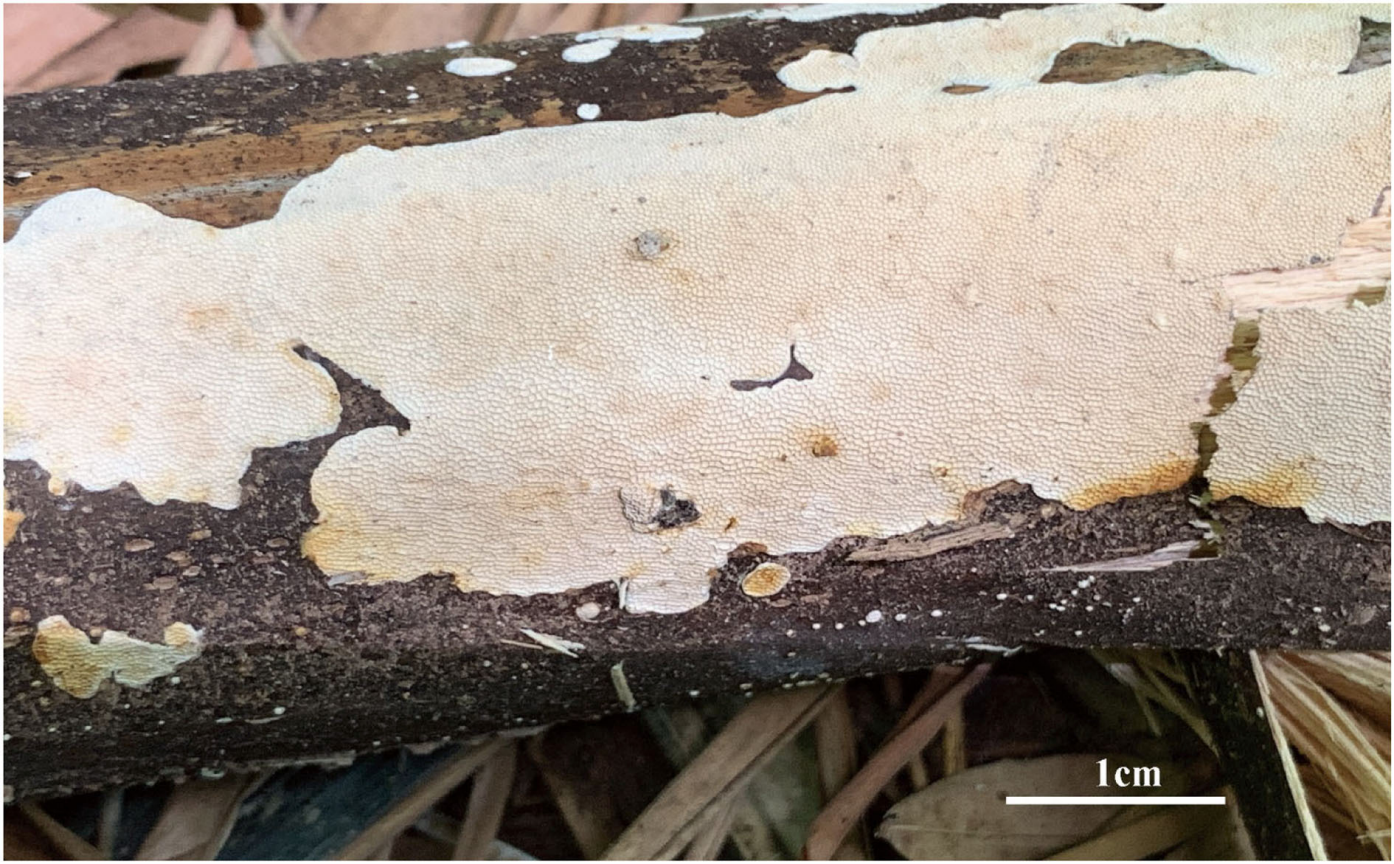
Basidiocarps of *Jorgewrightia tenuis* (holotype, Dai 20510).

**Figure 7:**
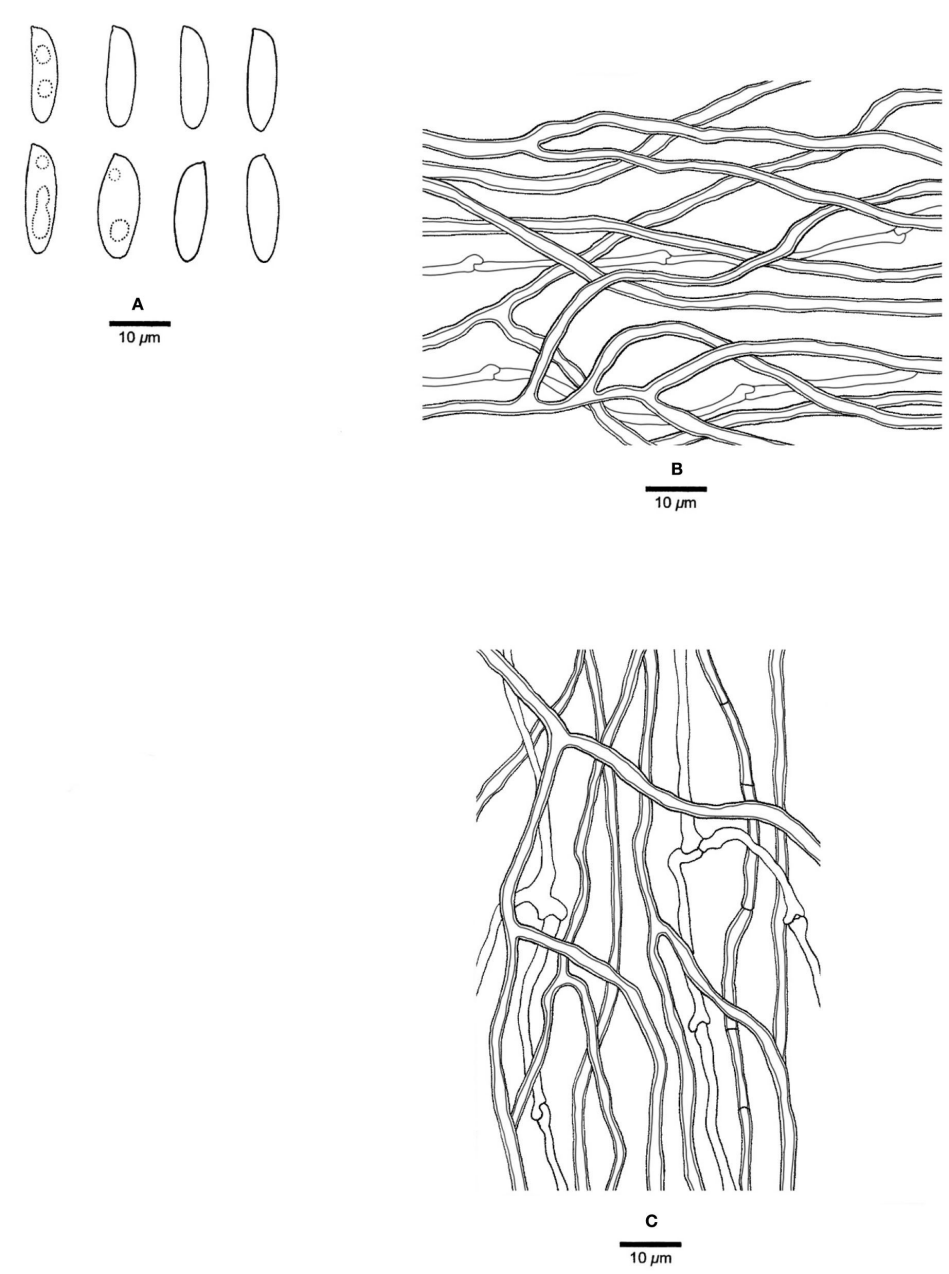
Microscopic structures of *Jorgewrightia tenuis* (drawn from holotype, Dai 20510). **(A)** basidiospores, **(B)** hyphae from subiculum, and **(C)** hyphae from tube trama.

***Jorgewrightia tenuis*** Y. C. Dai, Yuan Yuan, Ya R. Wang and Y. D. Wu, sp. nov. Figures 6, 7

MycoBank: 845313

*Type:* China, Yunnan Prov., Mengla County, Tropical Rain Forest Valley, on dead bamboo, 18 August 2019, Dai 20510 (holotype, BJFC032178!).

*Etymology: tenuis* (Lat.) refers to the extremely thin basidiocarps.

*Basidiocarps* annual, resupinate, adnate, corky, without odor or taste when fresh, becoming hard corky upon drying, up to 19 cm long, 3 cm wide, and 0.18 mm thick at the center; sterile margin very narrow to almost lacking. Pore surface cream when fresh, buff when dry; pores angular, 3–3.5 per mm; dissepiments thin, entire; subiculum pale cream, corky, extremely thin to almost absent; tubes cream, corky, up to 0.18 mm long. *Hyphal system* dimitic; generative hyphae with clamp connections; skeletal hyphae sometimes simple septate, weakly dextrinoid, CB+; tissues unchanged in KOH (not swollen). *Subicular* generative hyphae hyaline, thin-walled, unbranched, 1.5–1.8 μm diameter; skeletal hyphae dominant, thick-walled with a medium to wide lumen, moderately branched, flexuous, interwoven, 2–2.5 μm diameter. *Tramal* generative hyphae frequent, hyaline, thin-walled, moderately branched, 1.2–1.5 μm diameter; skeletal hyphae dominant, thick-walled with a medium to wide lumen, moderately branched, flexuous, interwoven, 1.5–2.5 μm diameter. *Dendrohyphidia* absent. *Hyphal pegs* absent. *Cystidia* absent; *cystidioles* absent. *Basidia* not seen. Small tetrahedric or polyhedric crystals frequent among hymenium and trama. *Basidiospores* fusiform, hyaline, thin-walled, smooth, sometimes with one or two small guttules, IKI–, CB–, (15–) 16.5–17 (−17.2) × (4.2–) 4.8–5.5 (−5.8) μm, *L* = 16.47 μm, *W* = 5.02 μm, *Q* = 3.28–3.42 (*n* = 60/2).

*Additional materials (paratypes) examined:* China, Yunnan Prov., Mengla County, Tropical Rain Forest Valley, on dead bamboo, 18 August 2019, Dai 20517 (BJFC032185!).

*Notes:* For the phylogenetic relationships of *Jorgewrightia tenuis* and other species, refer to the notes of *J. austroasiana*. Morphologically, *J. tenuis* resembles *J. bambusae* and *J. rimosa* by the adnate and extremely thin basidiocarps, but *J. bambusae* has thick-walled and ellipsoid basidiospores, and *J. rimosa* has dendrohyphidia (Yuan et al., 2017; Wang et al., 2021). In addition, *J. tenuis* is similar to *J. fusiformis*, both present with fusiform basidiospores, but *J. fusiformis* has indextrinoid skeletal hyphae and dendrohyphidia (Wang et al., 2021).

**Figure 8:**
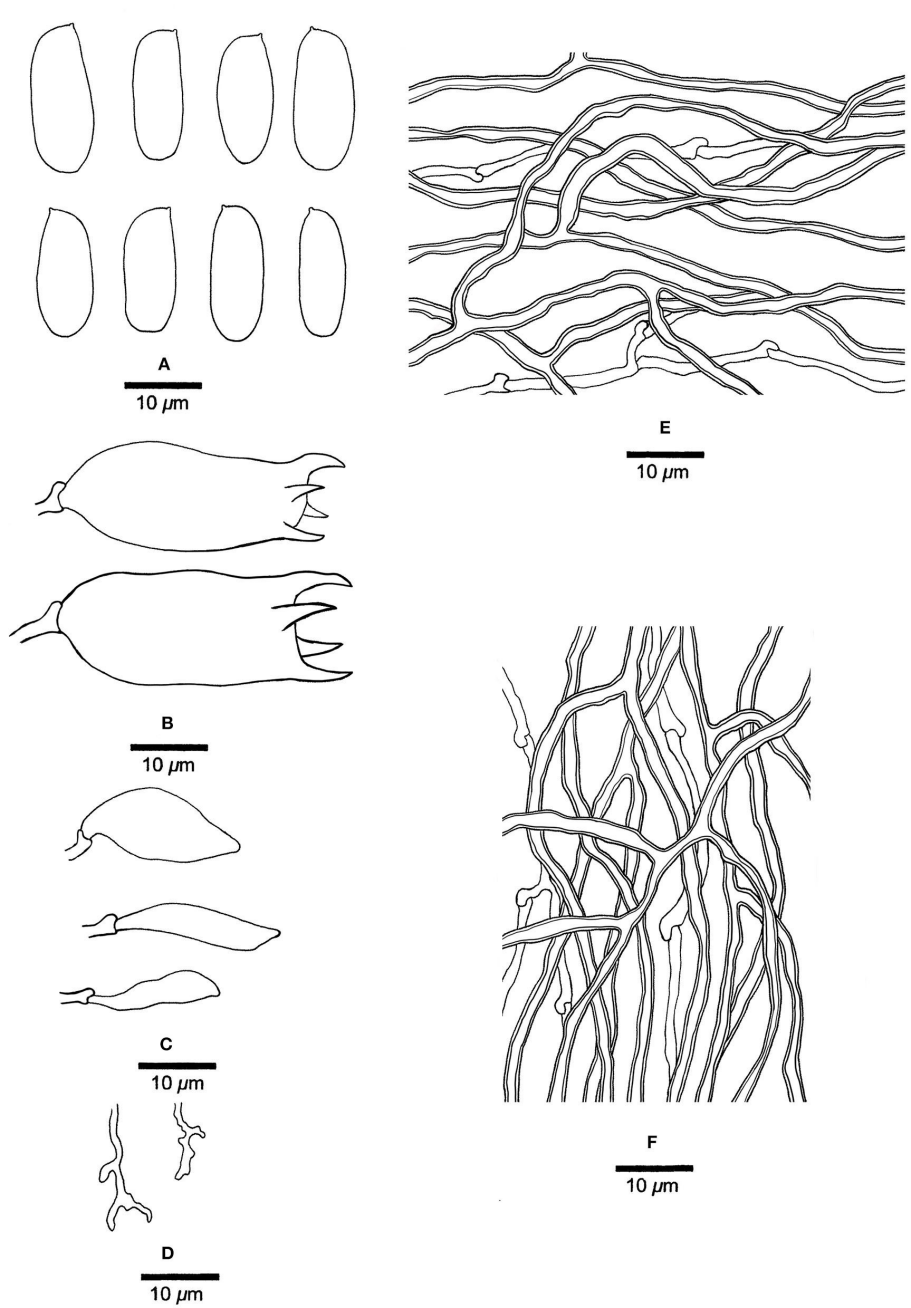
Microscopic structures of *Jorgewrightia kirkii* (drawn from Ryvarden 32577). **(A)** Basidiospores. **(B)** Basidia. **(C)** Cystidioles. **(D)** Dendrohyphidia. **(E)** Hyphae from subiculum. **(F)** Hyphae from tube trama.

***Jorgewrightia kirkii*** (Masuka and Ryvarden) Y. C. Dai, Yuan Yuan, Ya R. Wang and Y. D. Wu, comb. nov. Figure 8

MycoBank: 845318

Basionym: *Dichomitus kirkii* Masuka and Ryvarden, Mycological Research 103 (9): 1129 (1999).

*Basidiocarps* annual, resupinate, corky when dry, around 1 mm thick at the center; sterile margin very narrow to almost lacking. Pore surface clay buff to isabelline when dry; pores round, 1.5–2 per mm; dissepiments thin, entire; subiculum clay buff, corky, up to 0.2 mm thick; tubes concolorous with the pore surface, corky, up to 0.8 mm long. *Hyphal system* dimitic; generative hyphae with clamp connections; skeletal hyphae indextrinoid, CB+; tissues unchanged in KOH (not swollen). *Subicular* generative hyphae hyaline, thin-walled, occasionally branched, 1.5–1.8 μm diameter; skeletal hyphae dominant, thick-walled with a wide lumen, moderately branched, flexuous, interwoven, 2.5–3 μm diameter. *Tramal* generative hyphae, hyaline, thin-walled, moderately branched, 1.2–1.5 μm diameter; skeletal hyphae dominant, thick-walled with a wide lumen, frequently branched, flexuous, interwoven, 1.5–2.5 μm diameter. *Dendrohyphidia* present. *Hyphal pegs* absent. *Cystidia* absent; *cystidioles* present, fusoid to ventricose, thin-walled, smooth, 23–28 × 6.5–15 μm. *Basidia* barrel-shaped, with four sterigmata and a basal clamp connection, 30–40 × 16–18 μm; *basidioles* in shape similar to basidia. Small tetrahedric or polyhedric crystals frequent among hymenium and trama. *Basidiospores* cylindrical, hyaline, thin-walled, smooth, IKI–, CB–, (20.2–) 21–23.5 (−24) × (7–) 7.5–8.8 (−9) μm, *L* = 21.86 μm, *W* = 8.1 μm, *Q* = 2.70 (*n* = 30/1).

*Materials examined:* Zimbabwe, Mashonaland, Binga Forest East of Harare, on an angiosperm wood, 27 January 1993, Ryvarden 32577 (O, dupl. BJFC002897!); Ryvarden 33631 (holotype, O).

*Notes*: *Jorgewrightia kirkii* was originally described as *Dichomitus kirkii* Masuka and Ryvarden from Africa (Masuka and Ryvarden, 1999). It is extremely large basidiospores (21–23.5 × 7.5–8.8 μm) that are unique in *Megasporoporia* sensu lato. Our phylogeny (Figure 1) shows that the species is nested in *Jorgewrightia* clade with robust support (100% MP, 100% ML, 1.00 BPP). Hence, the above combination is proposed.

**Figure 9:**
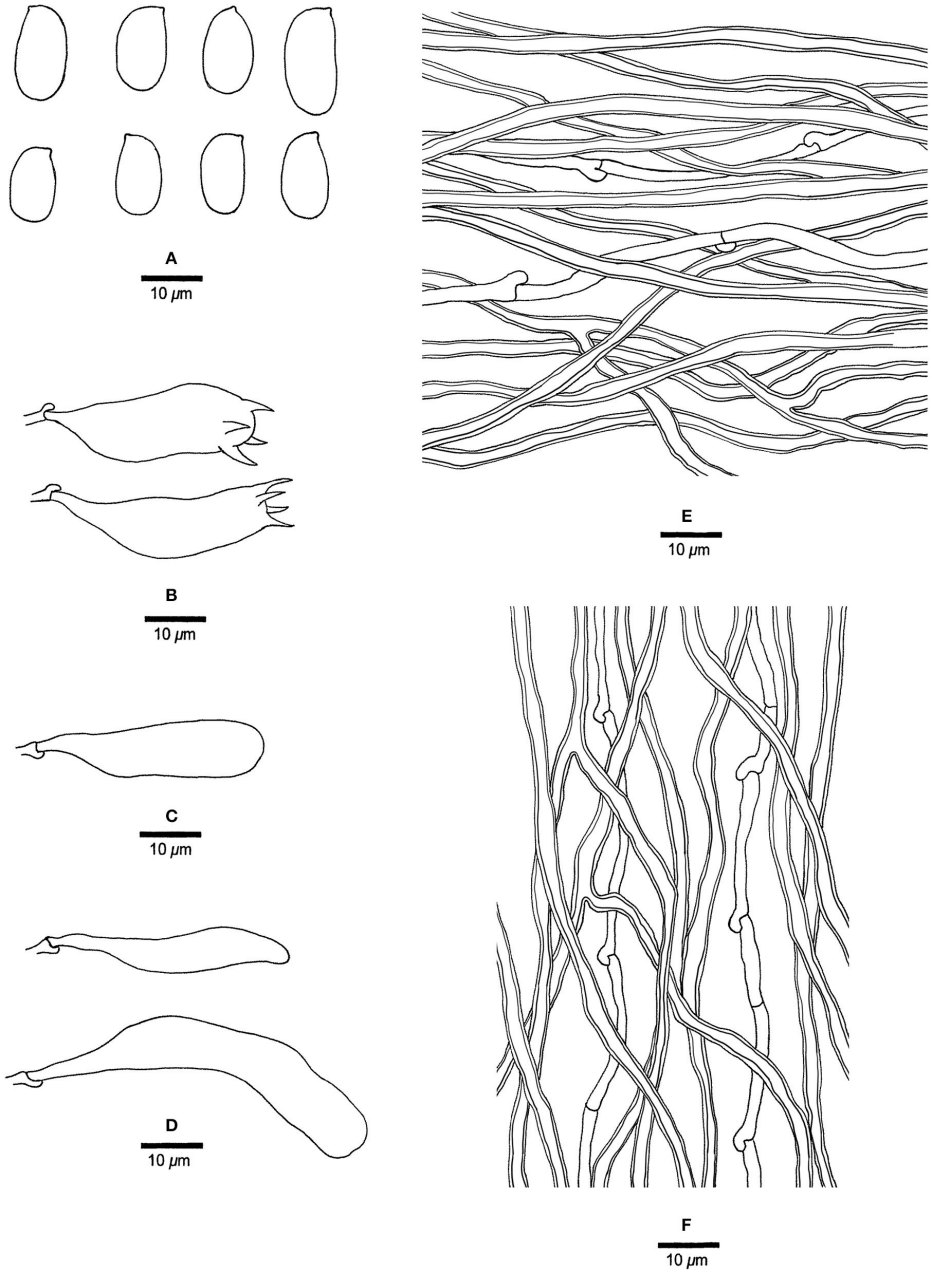
Microscopic structures of *Mariorajchenbergia epitephra* (drawn from Coveny 219). **(A)** Basidiospores. **(B)** Basidia. **(C)** A basidiole. **(D)** Cystidioles. **(E)** Hyphae from subiculum. **(F)** Hyphae from tube trama.

***Mariorajchenbergia epitephra*** (Berk.) Y. C. Dai, Yuan Yuan, Ya R. Wang and Y. D. Wu, comb. nov. Figure 9

MycoBank: 845319

Basionym: *Trametes epitephra* Berk., J. Linn. Soc., Bot. 13: 165 (1872).

= *Dichomitus epitephrus* (Berk.) Ryvarden, Mycotaxon 20 (2): 339 (1984).

*Basidiocarps* biennial, pileate, solitary, attached by a broad lateral base. Pilei ungulate, hard corky when dry, projecting up to 5 mm, 7 mm wide, and 5.5 mm thick at the base. Pore surface cream to buff when dry; pores round to sinuous, 1–2 per mm; dissepiments thick, entire; subiculum pale buff, corky, up to 0.5 mm thick; tubes concolorous with the pore surface, corky, up to 5 mm long. *Hyphal system* dimitic; generative hyphae with clamp connections; skeletal hyphae indextrinoid, CB+; tissues become slightly swollen in KOH. *Subicular* generative hyphae hyaline, thin-walled, unbranched, 1.8–2 μm diameter; skeletal hyphae dominant, thick-walled with a wide lumen, infrequently branched, flexuous, interwoven, 2–2.8 μm diameter. *Tramal* generative hyphae hyaline, thin-walled, unbranched, 1.8–2.5 μm diameter; skeletal hyphae dominant, thick-walled with a medium to wide lumen, infrequently branched, flexuous, interwoven, 2.5–3 μm diameter. *Dendrohyphidia* absent. *Hyphal pegs* present. *Cystidia* absent; *cystidioles* present, fusoid to clavate, thin-walled, smooth, 26.5–50.5 × 6–13.2 μm. *Basidia* clavate, with four sterigmata and a basal clamp connection, 28.5–35.5 × 7.5–10.2 μm; *basidioles* in shape similar to basidia, but slightly smaller. Small tetrahedric crystals frequent among hymenium and trama. *Basidiospores* broadly ellipsoid, hyaline, thin-walled, smooth, IKI–, CB–, 9.5–16.5 × 7–9 μm, *L* = 13.28 μm, *W* =7.8 μm, *Q* = 1.7 (*n* = 15/1).

*Material examined:* Australia, New South Wales, Blackett, on *Eucalyptus moluccana*, 10 July 1983, Coveny 219 (H, JV, dupl. BJFC002895!).

*Notes: Mariorajchenbergia epitephra* was originally described as *Trametes epitephra* from South Australia. Our studied sample fits the original description of *Trametes epitephra* (Berkeley, 1872; Cunningham, 1965). Phylogenetically, the species is nested in the *Mariorajchenbergia* clade. Therefore, the above combination is proposed. The species has pileate basidiocarps that are unique to *Megasporoporia* sensu lato.

***Mariorajchenbergia leucoplaca*** (Berk.) Y. C. Dai and P. K. Buchanan, comb. nov.

MycoBank: 845320

Basionym: *Polyporus leucoplacus* Berk., Fl. N. Zealand 2: 180 (1855).

= *Dichomitus leucoplacus* (Berk.) Ryvarden, Norweg. J. Bot. 24: 222 (1977).

= *Megasporoporiella australiae* Y. C. Dai, Yuan Yuan and Ya. R. Wang, in Wang, Wu, Vlasák, Yuan and Dai, Mycosphere 12 (1): 1027 (2021).

*Megasporoporiella australiae* was recently described in Australia (Wang et al., 2021). However, its vouchers and samples of *Polyporus leucoplacus* from New Zealand are nested together in a subclade with robust support in the *Mariorajchenbergia* clade. In addition, the morphology of these two taxa is similar (Wang et al., 2021), and the former becomes a synonym of the latter, with the above combination proposed.

*Materials examined:* Australia, Melbourne, Dandenong Ranges Botanic Garden, on a dead tree of *Rhododendron*, 12 May 2018, Y. C. Dai 18657 (BJFC027125!, holotype of *Megasporoporiella australiae*). New Zealand, Auckland, Waitakere Ranges, on a fallen wood, 11 April 1989, P. K. Buchanan 89/037 (ICMP 16412); Northland, William Hewett Reserve, on decaying wood, 2007, B. C. Paulus BCP3987 (ICMP 16962); Taupo, Kurua Reserve, Owhango, 3 Oct 2007, on a decaying branch (probably *Dacrydium cupressinum*), B. C. Paulus AOD348 (ICMP 17545).

**Figure 10:**
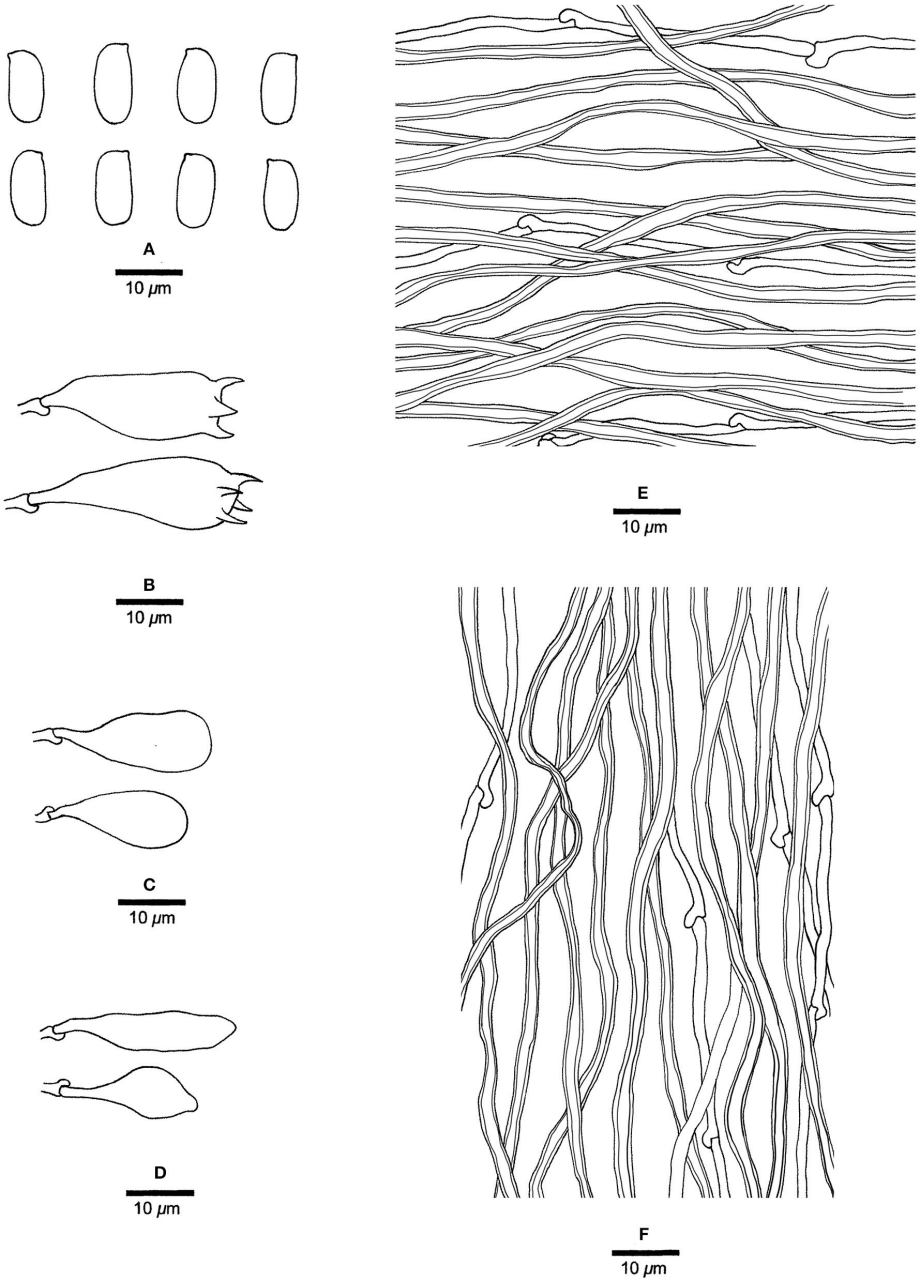
Microscopic structures of *Mariorajchenbergia subleucoplaca* (drawn from Ryvarden 11049). **(A)** Basidiospores. **(B)** Basidia. **(C)** Basidioles. **(D)** Cystidioles. **(E)** Hyphae from subiculum. **(F)** Hyphae from tube trama.

***Mariorajchenbergia subleucoplaca*** Y. C. Dai and P. K. Buchanan, sp. nov. Figure 10

MycoBank: 845315

*Type:* Tanzania, Morogoro, Uluguri Mts., Morning Side Nature Reserve, 24 February 1973, Ryvarden 11049 (holotype, O; isotype, BJFC002898!).

*Etymology: subleucoplaca* (Lat.) refers to the species that somewhat resemble *Mariorajchenbergia leucoplaca*.

*Basidiocarps* annual, resupinate, corky when dry, around 0.4 mm thick at the center; sterile margin distinct, white, up to 0.2 mm wide. Pore surface cream to buff when dry; pores round, 4–5 per mm; dissepiments thick, entire; subiculum pale cream, corky, up to 0.3 mm thick; tubes concolorous with the pore surface, corky, up to 0.1 mm long. *Hyphal system* dimitic; generative hyphae with clamp connections; skeletal hyphae indextrinoid, CB+; tissues unchanged in KOH (not swollen). *Subicular* generative hyphae infrequent, hyaline, thin-walled, unbranched, 1.2–1.8 μm diameter; skeletal hyphae dominant, thick-walled with narrow to wide lumen, unbranched, flexuous, interwoven, 2.5–3 μm diameter. *Tramal* generative frequent, hyphae hyaline, thin-walled, unbranched, 1.2–1.5 μm diameter; skeletal hyphae dominant, thick-walled with a medium to wide lumen, unbranched, interwoven, 2.5–3.5 μm diameter. *Dendrohyphidia* absent. *Hyphal pegs* absent. *Cystidia* absent; *cystidioles* present, subulate or ventricose, thin-walled, smooth, 22.5–26.5 × 5.5–13.5 μm. *Basidia* more or less pyriform, with four sterigmata and a basal clamp connection, 23.2–26.5 × 9.5–11 μm; *basidioles* in shape similar to basidia, but distinctly smaller. Small tetrahedric or polyhedric crystals frequent among hymenium and trama. *Basidiospores* oblong ellipsoid, hyaline, thin-walled, smooth, IKI–, CB–, (10–) 11–12 × (4.5–) 5–6 μm, *L* = 10.94 μm, *W* = 5.2 μm, *Q* = 2.10 (*n* = 30/1).

*Notes: Mariorajchenbergia leucoplaca* was originally described as *Polyporus leucoplacus* from New Zealand [Berkeley, 1855; = *Dichomitus leucoplacus* (Berk.) Ryvarden (Ryvarden, 1977)]. Masuka and Ryvarden (1999) identified Tanzanian samples as *D. leucoplacus*. Our studied specimen (Ryvarden 11049) was also collected in Tanzania, and phylogenetically (Figure 1) is nested in the *Mariorajchenbergia* clade with robust support (53% MP, 91% ML, 0.98 BPP). Samples labeled as *D. leucoplaca* from Africa and New Zealand, therefore, nested into two independent lineages. Thus, we describe the African samples as a new species. It differs from *M.leucoplaca* by its smaller pores (4–5 per mm vs. 2–4 per mm) and shorter basidiospores (11–12 × 5–6 μm vs. 11.8–15 × 4–6 μm, Wang et al., 2021).

**Figure 11:**
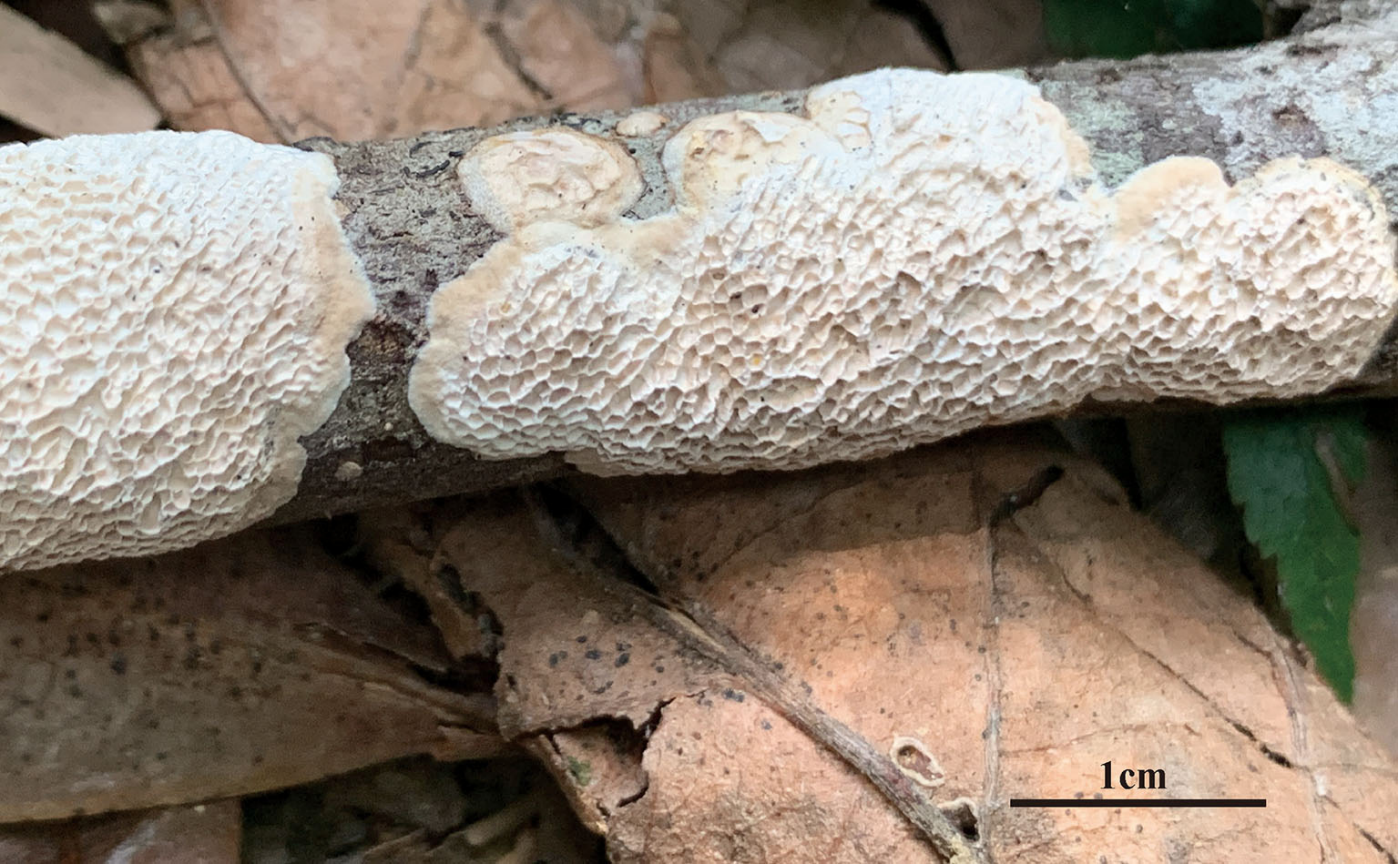
Basidiocarps of *Megasporia sinuosa* (holotype, Dai 22210).

**Figure 12:**
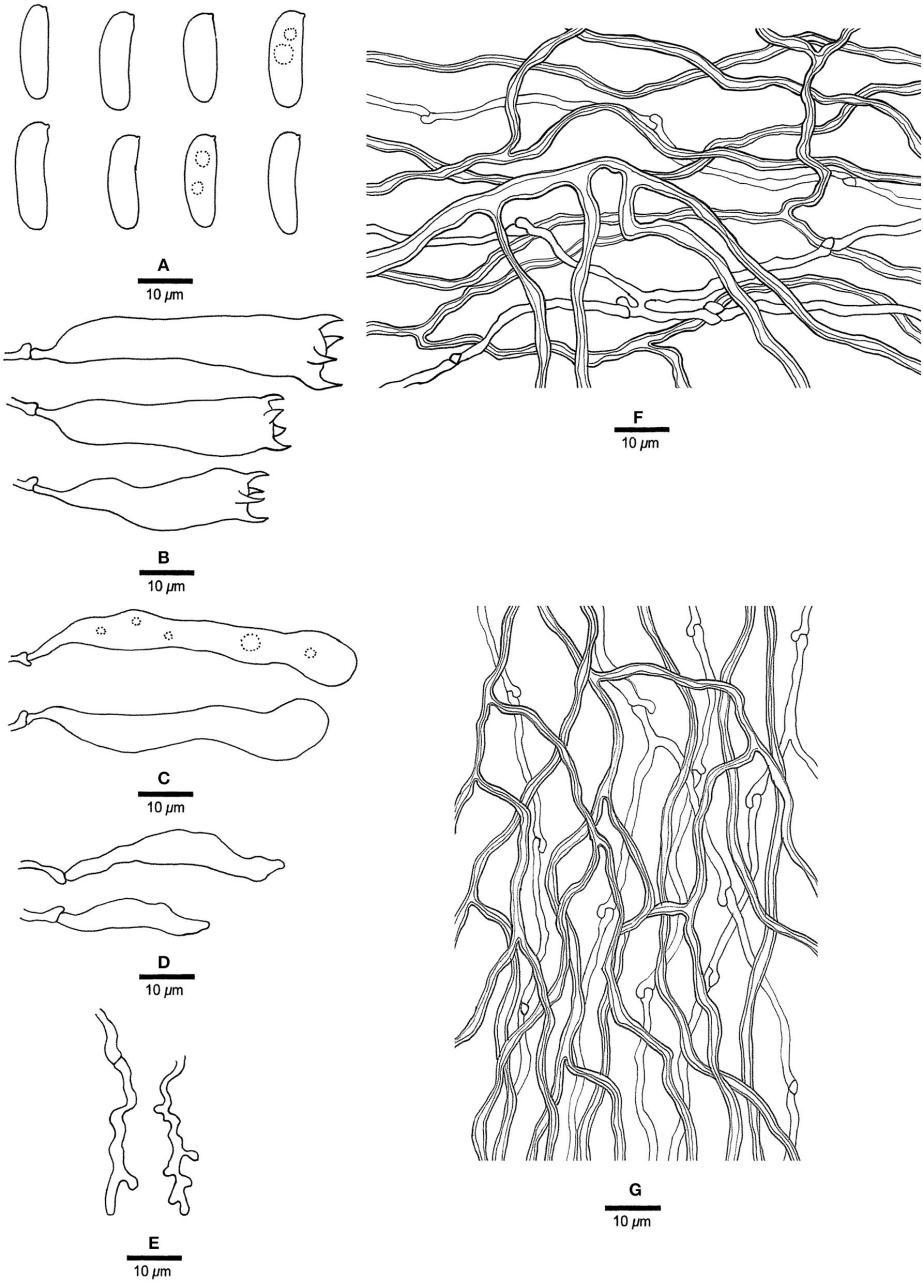
Microscopic structures of *Megasporia sinuosa* (drawn from holotype, Dai 22210). **(A)** Basidiospores. **(B)** Basidia. **(C)** Basidioles. **(D)** Cystidioles. **(E)** Dendrohyphidia. **(F)** Hyphae from subiculum. **(G)** Hyphae from tube trama.

***Megasporia sinuosa*** Y. C. Dai, Yuan Yuan, Ya R. Wang and Y. D. Wu, sp. nov. Figures 11, 12

MycoBank: 845316

*Type:* China, Hainan Prov., Qiongzhong County, Limushan Forest Park, on a fallen angiosperm branch, 31 March 2021, Dai 22210 (holotype, BJFC036801!).

*Etymology: sinuosa* (Lat.) refers to the species having sinuous pores.

*Basidiocarps* annual, resupinate, adnate, corky, without odor or taste when fresh, becoming hard corky upon drying, up to 3 cm long, 2 cm wide, and 0.6 mm thick at the center; sterile margin distinct, pale buff, up to 1.5 mm wide. Pore surface white to cream when fresh, cream to buff when dry; pores angular to sinuous, 1.5–2 per mm; dissepiments thick, entire to lacerate; subiculum pale buff, corky, up to 0.3 mm thick; tubes cream, corky, up to 0.3 mm long. *Hyphal system* dimitic; generative hyphae with clamp connections; skeletal hyphae strongly dextrinoid, CB+, slightly swollen in KOH. *Subicular* generative hyphae hyaline, thin-walled, occasionally branched, 1.5–2 μm diameter; skeletal hyphae dominant, thick-walled with a narrow to medium lumen, frequently branched, strongly flexuous, interwoven, 1.8–2.5 μm diameter. *Tramal* generative hyphae frequent, hyaline, thin-walled, moderately branched, 1.5–1.8 μm diameter; skeletal hyphae dominant, thick-walled with a narrow to medium lumen, frequently branched, strongly flexuous, interwoven, 1.5–2.8 μm diameter. *Dendrohyphidia* present. *Hyphal pegs* absent. *Cystidia* absent; *cystidioles* present, subulate or ventricose, thin-walled, smooth, 34.5–38.5 × 4.5–6.5 μm. *Basidia* clavate, usually constricted in middle, with four sterigmata and a basal clamp connection, 20–50.2 × 7–9.2 μm; *basidioles* similar to basidia, sometimes with a few guttules. Small tetrahedric or polyhedric crystals frequent among hymenium and trama. *Basidiospores* cylindrical to allantoid, hyaline, thin-walled, smooth, sometimes with one to two small guttules, IKI–, CB–, (15–) 15.2–16.8 (−17.2) × (4.5–) 4.8–5 μm, *L* = 16.3 μm, *W* = 4.87 μm, *Q* = 3.4 (*n* = 30/1).

*Additional materials (paratypes) examined:* China, Hainan Prov., Lingshui County, Diaoluoshan Forest Park, on a fallen angiosperm branch, 8 November 2020, Dai 22010 (BJFC035906!), Dai 22011 (BJFC035907!).

*Notes:* Phylogenetically, *Megasporia sinuosa* is closely related to *M. olivacea* (Figure 1), but the latter differs from the former by darker pore surface (deep olive vs. cream to buff), presence of hyphal pegs, and wider basidiospores (5.5–6.5 μm vs. 4.8–5 μm).

**Figure 13:**
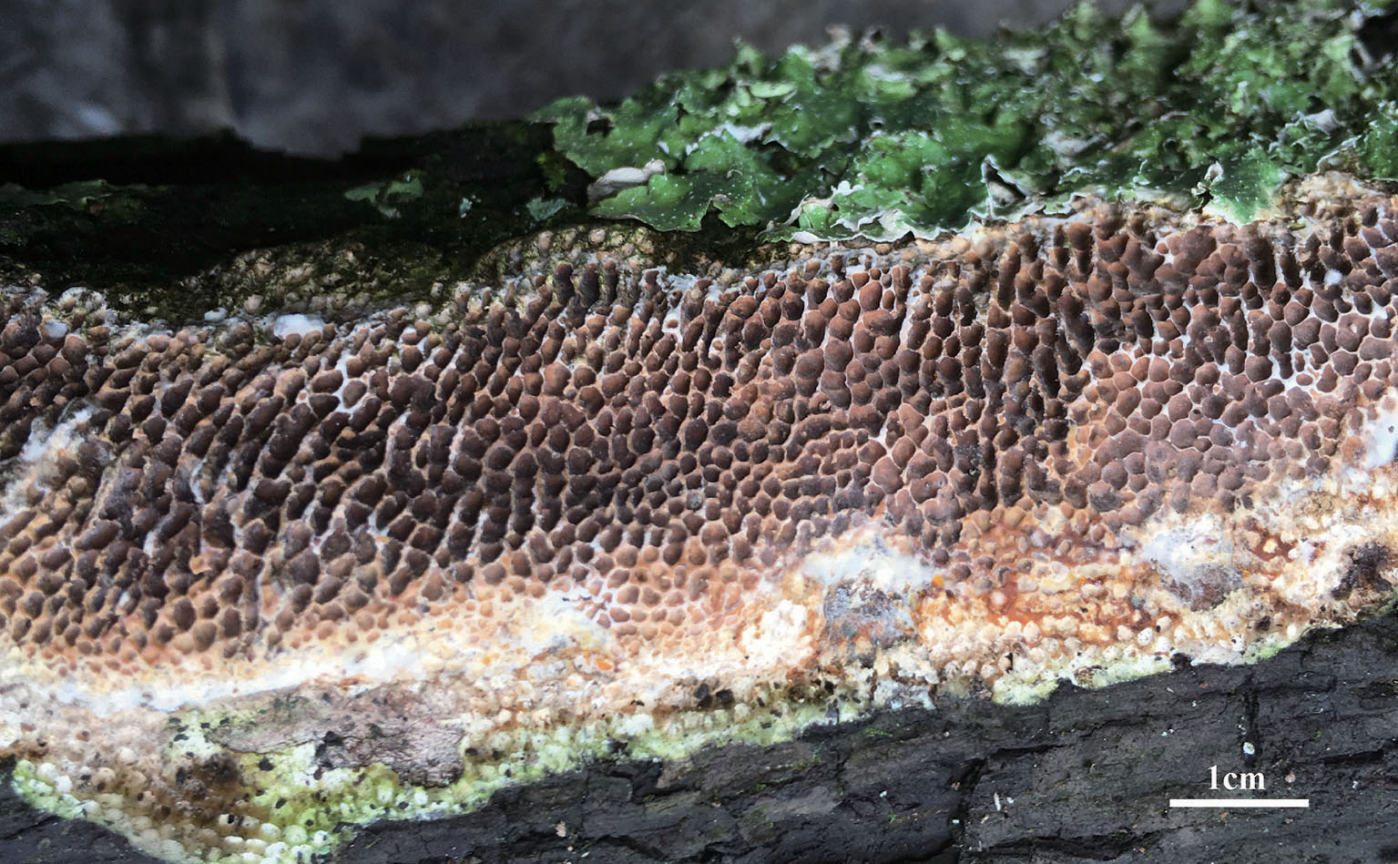
A basidiocarp of *Megasporia olivacea* (holotype, Dai 17908).

**Figure 14:**
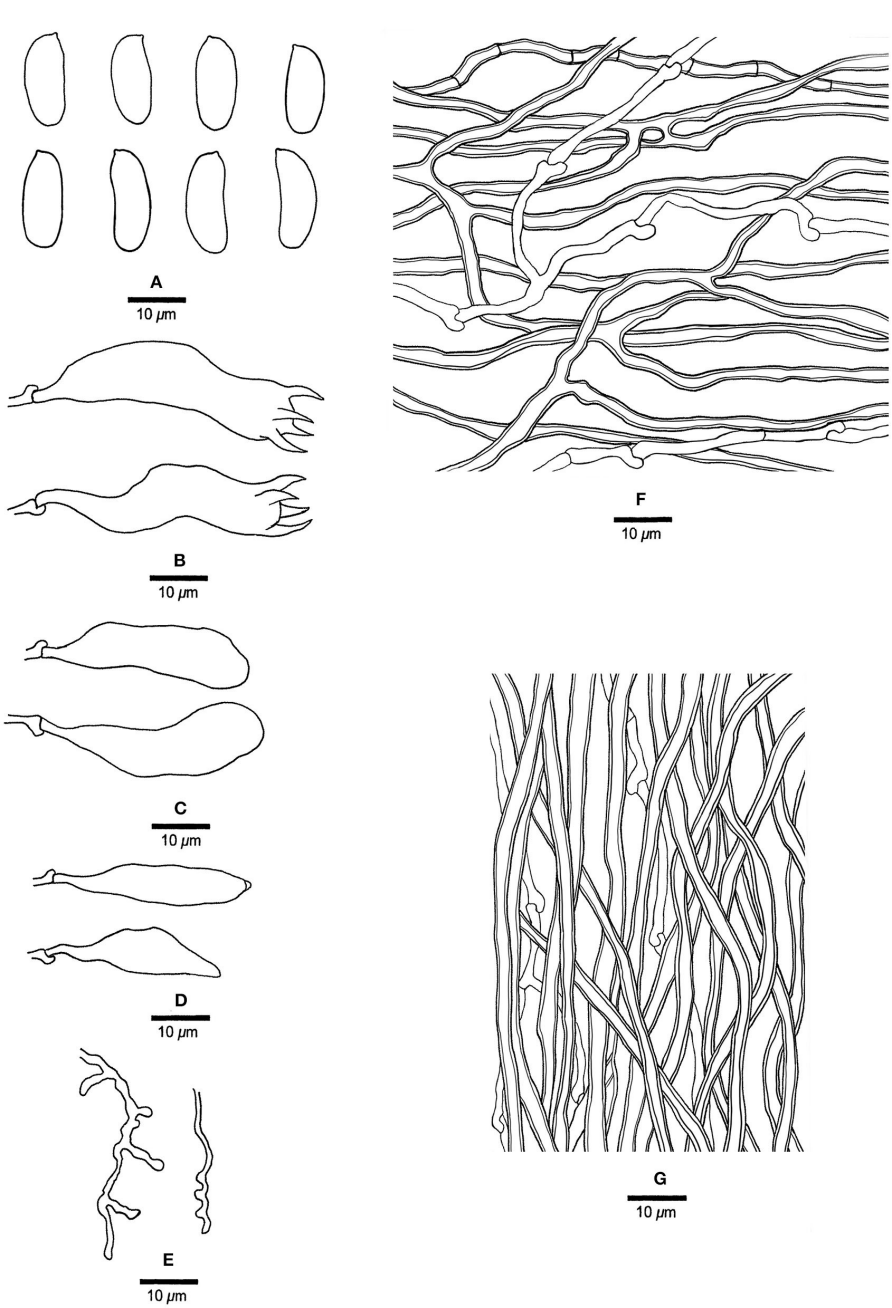
Microscopic structures of *Megasporia olivacea* (drawn from holotype, Dai 17908). **(A)** Basidiospores. **(B)** Basidia. **(C)** Basidioles. **(D)** Cystidioles. **(E)** Dendrohyphidia. **(F)** Hyphae from subiculum. **(G)** Hyphae from tube trama.

***Megasporia olivacea*** Y. C. Dai, Yuan Yuan, Ya R. Wang and Y. D. Wu, sp. nov. Figures 13, 14

MycoBank: 845317

*Type:* China, Hubei Prov., Wufeng County, Chaibuxi Geopark, on the dead tree of *Quercus*, 14 August 2017, Dai 17908 (holotype, BJFC025437!).

*Etymology: olivacea* (Lat.), refers to the species with a deeply olivaceous pore surface when dry.

*Basidiocarps* annual, resupinate, adnate, hard corky, without odor or taste when fresh, becoming hard corky upon drying, up to 14 cm long, 4.5 cm wide, and 4 mm thick at the center; sterile margin distinct, cream, up to 1.5 mm wide. Pore surface white to cream when fresh, deep olive when dry; pores angular, 0.5–1 per mm; dissepiments thin, entire; subiculum grayish brown, hard corky, up to 0.2 mm thick; tubes grayish buff, hard corky, up to 3.8 mm long. *Hyphal system* dimitic; generative hyphae with clamp connections; skeletal hyphae sometimes simple septate, moderately dextrinoid, CB+, strongly swollen in KOH. *Subicular* generative hyphae hyaline, thin-walled, occasionally branched, 1.8–2.5 μm diameter; skeletal hyphae dominant, thick-walled with a narrow to medium lumen, moderately branched, flexuous, interwoven, 2–3.5 μm diameter. *Tramal* generative hyphae hyaline, thin-walled, occasionally branched, 2–2.5 μm diameter; skeletal hyphae dominant, thick-walled with a narrow to wide lumen, unbranched, flexuous, interwoven, 2–3 μm diameter. *Dendrohyphidia* present. *Hyphal pegs* present. *Cystidia* absent. *Cystidioles* present, subulate or ventricose, thin-walled, smooth, 26–31 × 5.5–6.5 μm. *Basidia* clavate, usually constricted in middle, with four sterigmata and a basal clamp connection, 38–44 × 9–10 μm; *basidioles* in shape similar to basidia, but distinctly smaller. Small tetrahedral crystals frequent among hymenium and trama. *Basidiospores* cylindrical, some slightly curved, hyaline, thin-walled, smooth, IKI–, CB–, (14.5–) 15.2–17 (−18.2) × (4.5–) 5.5–6.5 (−7) μm, *L* = 16.4 μm, *W* = 5.69 μm, *Q* = 2.73–2.95 (*n* = 60/2).

*Additional materials (paratypes) examined:* China, Hubei Prov., Wufeng County, Chaibuxi Geopark, on a dead tree of *Quercus*, 14 August 2017, Dai 17909 (BJFC025438!).

*Notes:* Morphologically, *Megasporia olivacea* has a deep olive pore surface when dry which is unique to *Megasporoporia* sensu lato.

**Figure 15:**
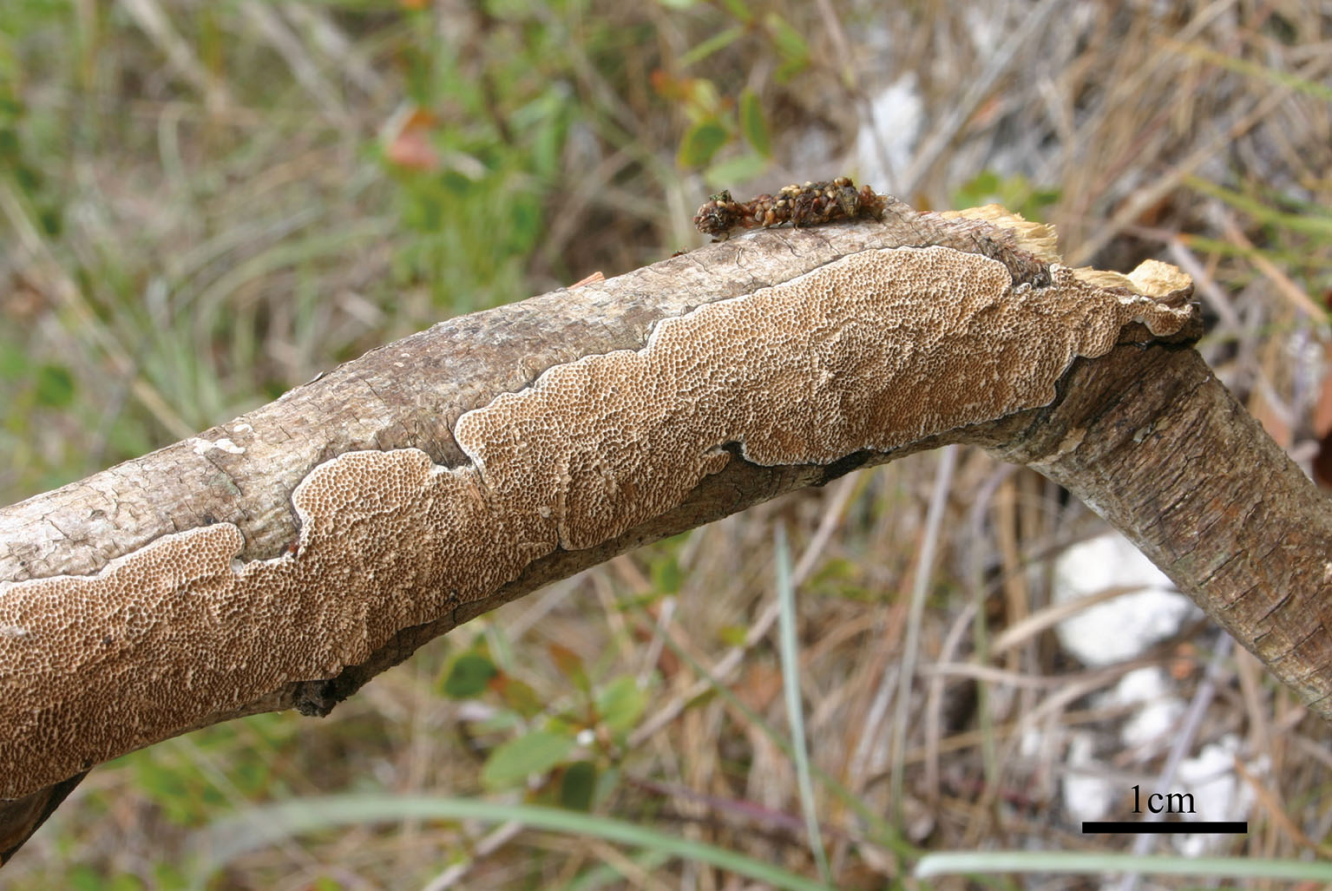
A basidiocarp of *Megasporia* sp. 1 (JV 0904/50-J).

**Figure 16:**
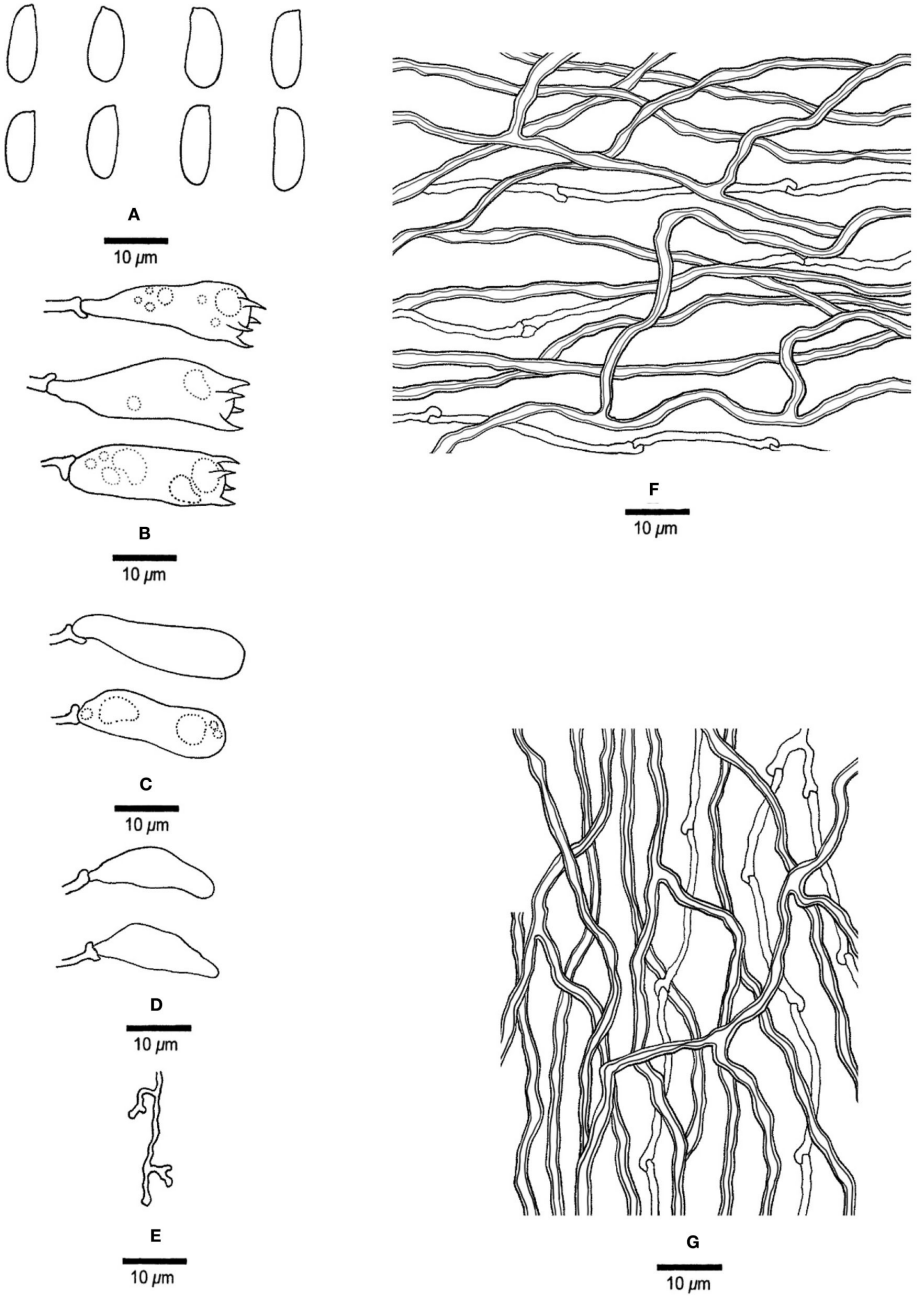
Microscopic structures of *Megasporia* sp. 1 (drawn from JV 0904/52-J). **(A)** basidiospores, **(B)** basidia, **(C)** basidioles, **(D)** cystidioles, **(E)** dendrohyphidia, **(F)** hyphae from subiculum, and **(G)** hyphae from tube trama.

*Megasporia* sp. 1 Figures 15, 16

*Basidiocarps* annual, resupinate, adnate, corky, without odor or taste when fresh, becoming hard corky upon drying, up to 8 cm long, 3 cm wide, and 0.6 mm thick at the center; sterile margin indistinct, white, very narrow to almost absent with age. Pore surface pale ochraceous when fresh and dry; pores mostly angular, 2–3 per mm; dissepiments thin, lacerate; subiculum cream, corky, up to 0.2 mm thick; tubes concolorous with the pore surface, corky, up to 0.4 mm long. *Hyphal system* dimitic; generative hyphae with clamp connections; skeletal hyphae strongly dextrinoid, CB+, strongly swollen in KOH. *Subicular* generative hyphae hyaline, thin-walled, unbranched, 1.8–2 μm diameter; skeletal hyphae dominant, thick-walled with a narrow to medium lumen, moderately branched, flexuous, interwoven, 2–2.5 μm diameter. *Tramal* generative hyphae hyaline, thin-walled, occasionally branched, 1.8–2 μm diameter; skeletal hyphae dominant, thick-walled with a narrow to medium lumen, moderately branched, strongly flexuous, interwoven, 2–3 μm diameter. *Dendrohyphidia* present. *Hyphal pegs* absent. *Cystidia* absent; *cystidioles* present, fusiform, thin-walled, smooth, 18–23 × 5–6 μm. *Basidia* pyriform to barrel-shaped, with four sterigmata and a basal clamp connection, usually with a few small guttules, 34.5–28.5 × 7.8–10.2 μm; *basidioles* in shape similar to basidia, but smaller. Small polyhedric crystals frequently present among hymenium and trama. *Basidiospores* cylindrical, hyaline, thin-walled, smooth, IKI–, CB–, (10.5–) 12.5–14.2 (−15) × (4.2–) 4.5–4.8 (−5) μm, *L* = 12.98 μm, *W* = 4.58 μm, *Q* = 2.76–3.09 (*n* = 30/2).

*Materials examined:* USA, Florida, Long Pine Key, April 2009, JV 0904/50-J (JV, BJFC038548!), JV 0904/52-J (JV, BJFC038549!).

*Notes*: *Megasporoporia hexagonoides* was originally described as *Poria hexagonoides* Speg. from Argentina (Spegazzini, 1898), and a detailed description was given by Ryvarden et al. (1982). Lira et al. (2021) analyzed the nLSU sequence of the Argentine specimen (CBS 464.63) and treated it as *M. hexagonoides*. Our studied samples from Florida, USA have the same nLSU sequence as CBS 464.63, but their morphological characteristics are very different from that of *M. hexagonoides* (the latter has pores 0.5–1 per mm, absence of dendrohyphidia, basidiospores 16.6–21.8 × 5.2–6.8 μm, Ryvarden et al., 1982).

*Poria linearis* Murrill, described from Panama (Murrill, 1920), was treated as a synonym of *Megasporia cavernulosa* (Berk.) C. R. S. Lira and T. B. Giberton (=*Megasporoporia cavernulosa*), described from Panuré, Brazil (Berkeley, 1856). The morphology of the taxon from Florida fits *Poria linearis* well, but so far no DNA data are available from the Panamanian sample, and for the time being, we treat the Florida samples as *Megasporia* sp. 1.

## Key to known species of *Megasporoporia* sensu lato

1. Pores < 1 per mm………………………………………. 2

1. Pores > 1 per mm………………………………………. 5

2. Dendrohyphidia present…………………………………3

2. Dendrohyphidia absent…………………………………. 4

3. Hyphal pegs absent…………………*Jorgewrightia irregularis*

3. Hyphal pegs present……………………*Megasporia olivacea*

4. Basidiospores > 20 μm long………….*Megasporia mexicana*

4. Basidiospores < 20 μm long…..*Mariorajchenbergia epitephra*

5. Pores 5–7 per mm………………………………………..6

5. Pores 1–5 per mm………………………………………..7

6. Pore surface violet to greyish violet……*Jorgewrightia violacea*

6. Pore surface cream to buff………….. *Megasporoporia minor*

7. Basidiospores ellipsoid……………………………………8

7. Basidiospores cylindrical, allantoid, or fusiform……….…13

8. Pores 4–5 per mm………………………………………..9

8. Pores 1–2 per mm………………………………………11

9. Basidiospores thick-walled…………*Jorgewrightia bambusae*

9. Basidiospores thin-walled……………….………………10

10. Skeletal hyphae dextrinoid……………….….….…………

…………………….….….…*Mariorajchenbergia rhododendri*

10. Skeletal hyphae indextrinoid………………………………

……………………………*Mariorajchenbergia subleucoplaca*

11. Basidiospores > 15 μm long; pore surface pale purplish brown………………………………*Megasporia anoectopora*

11. Basidiospores < 15 μm long; pore surface cream to yellow.12

12. Pores 1–1.5 per mm; hyphal pegs present……….…………

…..….………………………………*Jorgewrightia ellipsoidea*

12. Pores 2 per mm; hyphal pegs absent………………….……

………………………………………*Megasporia amazonica*

13. Basidiospores fusiform…………………………………14

13. Basidiospores cylindrical or allantoid….….….…………15

14. Dendrohyphidia present; skeletal hyphae indextrinoid.….…

…….….….….………………………*Jorgewrightia fusiformis*

14. Dendrohyphidia absent; skeletal hyphae weakly dextrinoid...

……………………………………….…*Jorgewrightia tenuis*

15. Hyphal pegs present……………………………………16

15. Hyphal pegs absent………………………….…………21

16. Cystidioles present………………………….….………17

16. Cystidioles absent…………………………….….….…19

17. Basidiospores > 15 μm long….….….…*Jorgewrightia major*

17. Basidiospores < 15 μm long……………………………18

18. Basidiospores cylindrical………*Megasporoporia bannaensis*

18. Basidiospores allantoid……………………………………

…………………….…*Mariorajchenbergia pseudocavernulosa*

19. Dendrohyphidia present…………………………….….…

…….….…………………*Mariorajchenbergia subcavernulosa*

19. Dendrohyphidia absent……………………….….….…20

20. Basidiospores 3–4 μm wide; American species……….….…

……………….…………………*Megasporoporia neosetulosa*

20. Basidiospores 4.2–5.7 μm wide; African species.….….….…

….….….……………………………*Megasporoporia setulosa*

21. Dendrohyphidia present………………….….…………22

21. Dendrohyphidia absent…………….….…….…………28

22. Skeletal hyphae indextrinoid……………………………23

22. Skeletal hyphae weakly to strongly dextrinoid….….….…24

23. Basidiocarps annual; basidiospores > 20 μm long…………

….….….…………………………………*Jorgewrightia kirkii*

23. Basidiocarps biennial; basidiospores < 20 μm long.….….…

….……………………………*Mariorajchenbergia hubeiensis*

24. Skeletal hyphae strongly dextrinoid….….………………25

24. Skeletal hyphae weakly dextrinoid….….….….…………27

25. Cystidioles absent…………………*Megasporia cavernulosa*

25. Cystidioles present…………………………….….….…26

26. Basidiospores > 15 μm long; Asian species….….….………

…….……………………………………*Megasporia sinuosa*

26. Basidiospores < 15 μm long; North American species.….…

…………………………….….……………*Megasporia* sp. 1

27. Basidiocarps cracked when dry…….…*Jorgewrightia rimosa*

27. Basidiocarps uncracked when dry……….….….….………

……………………………………*Jorgewrightia yunnanensis*

28. Skeletal hyphae indextrinoid……………………………29

28. Skeletal hyphae moderately to strongly dextrinoid………30

29. Basidiospores > 15 μm long……*Jorgewrightia austroasiana*

29. Basidiospores < 15 μm long………………….….….….…

………………………………*Mariorajchenbergia leucoplaca*

30. Cystidioles absent…………….….….…………………31

30. Cystidioles present….….………………………………32

31. Basidiospores 10–11.8 μm long; Asian species……….….…

………………………………………*Megasporoporia inflata*

31. Basidiospores 12–13 μm long; South American species……

……………………………………*Megasporia variabilicolor*

32. Skeletal hyphae strongly dextrinoid……………….….…33

32. Skeletal hyphae moderately dextrinoid…………………34

33. Pores 2–3 per mm……………………*Jorgewrightia tropica*

33. Pores 4–5 per mm…….….….*Jorgewrightia guangdongensis*

34. Basidiospores 16–21 μm long……*Megasporia hexagonoides*

34. Basidiospores < 15 μm long……………………………35

35. Pore surface cream to buff, pores 2–3 per mm….….………

…………………………………*Jorgewrightia hengduanensis*

35. Pore surface pale pinkish-brown to salmon, pores 3–5 per

mm ……………………………*Jorgewrightia cystidiolophora*.

## Discussion

*Megasporoporia* was established based on *M. setulosa* (the type from Tanzania), but the type has probably been lost (Ryvarden et al., 1982). Lira et al. (2021) analyzed sequences from an African specimen (LR 9907), providing the most reliable molecular data for the species, conforming to the *Megasporoporia* sensu stricto clade. An additional three species were included in *Megasporoporia*: *M. cavernulosa* (type from Brazil)*, M. hexagonoides* (type from Argentina), and *M. mexicana* (type from Mexico). Phylogenetically these three are nested in another clade (*Megasporia* clade, Lira et al., 2021).

To date, four genera, *Jorgewrightia, Mariorajchenbergia, Megasporia*, and *Megasporoporia* sensu stricto, which include 36 species are accepted in *Megasporoporia* sensu lato.

The type of *Megasporia cavernulosa* was from Brazil, and the sequences KX584458 and KX619582 are from the Brazilian specimen URM 83867. Lira et al. (2021) considered this specimen to represent *M. cavernulosa*. This species was also reported from Florida, USA, and the morphology of our studied Florida samples (JV 0904/81-J, JV 0904/50-J, and JV 0904/52-J) is consistent with the description of *M. cavernulosa*. However, phylogenetically, these samples are distantly related to URM 83867. So, the occurrence of *M. cavernulosa* in the USA is uncertain.

*Dichomitus amazonicus* Gomes-Silva et al. was described from Amazonas (Gomes-Silva et al., 2012) and was later combined as *Megasporia amazonica* (Gomes-Silva et al.) C. R. S. Lira and Gibertoni (Lira et al., 2021). The molecular data of the type specimen of *M. amazonica* (Ryvarden 48295) are not available. Its phylogenetic analysis was based on specimens URM 85601 (Brazil-Pernambuco) and URM 87859 (Brazil-Bahia), but these two specimens did not cluster together (Lira et al., 2021). We studied a part of URM 87859 and found that it has a dimitic hyphal system, strongly dextrinoid and CB+ skeletal hyphae, and ellipsoid to subcylindrical basidiospores, (10–) 10.2–11.5 (−12.2) × (4.2–) 4.5–5 μm. These characteristics are consistent with the original description of *M. amazonica*. Therefore, we believe that URM 87859 represents *M. amazonica*, and URM 85601 is treated as “*M. amazonica*” in our phylogeny (Figure 1).

Lira et al. (2021) demonstrated that *Dichomitus cylindrosporus* (Ryvarden 45186) is nested in the *Megasporia* clade, and they combined it as *Megasporia cylindrospora* (Ryvarden) C. R. S. Lira and Gibertoni. However, we studied the type (Ryvarden 44248) of *D. cylindrosporus* and failed to extract the DNA. Morphologically, we found that Ryvarden 44248 has hyphal pegs and pores 2.5–3 per mm, while Ryvarden 45186 lacks hyphal pegs and has pores 1.5–2 per mm. So, Ryvarden 45186 most probably does not represent *D. cylindrosporus*, and the phylogenetic relationship between *D. cylindrosporus* and *Megasporoporia* sensu lato is uncertain.

*Megasporoporia, Megasporia, Jorgewrightia, Mariorajchenbergia*, and other genera (*Dichomitus, Perenniporia, Crassisporus, Daedaleopsis, Datronia, Neodatronia, Polyporus*, etc.) are nested together with robust support in our phylogeny based on our selected samples (Figure 1). *Megasporia* and *Jorgewrightia* are related to each other with moderate support. These two genera and *Megasporoporia* and *Mariorajchenbergia* seem to be strikingly unrelated to each other although the four genera share a very similar morphology. We currently cannot resolve this dilemma, which requires additional genomic data and further analyses.

## Data availability statement

The datasets presented in this study can be found in online repositories. The names of the repository/repositories and accession number(s) can be found in the article/supplementary material.

## Author contributions

Y-RW, YY, and Y-CD: design of the research. Y-RW: performance of the research. Y-RW, Y-CD, YY, and Y-DW: data analysis and interpretation. Y-RW, H-GL, JV, Y-CD, YY, Y-DW, and PB: the collection of materials. Y-RW, Y-CD, YY, Y-DW, and PB: writing and revising the manuscript. All authors contributed to the article and approved the submitted version.
